# Decreased expression of connective tissue growth factor in non-small cell lung cancer is associated with clinicopathological variables and can be restored by epigenetic modifiers

**DOI:** 10.1007/s00432-016-2195-3

**Published:** 2016-07-08

**Authors:** Hanna Drzewiecka, Bartłomiej Gałęcki, Donata Jarmołowska-Jurczyszyn, Andrzej Kluk, Wojciech Dyszkiewicz, Paweł P. Jagodziński

**Affiliations:** 1 Department of Biochemistry and Molecular Biology, Poznan University of Medical Sciences, Święcickiego 6 Street, 60-781 Poznan, Poland; 2 Department of Thoracic Surgery, Poznan University of Medical Sciences, Szamarzewskiego 62 Street, 60-569 Poznan, Poland; 3 Department of Clinical Pathomorphology, Poznan University of Medical Sciences, Przybyszewskiego 49 Street, 60-355 Poznan, Poland

**Keywords:** Connective tissue growth factor (CTGF), Non-small cell lung cancer (NSCLC), Clinicopathological features, Epigenetic regulation, 5-Aza-2′-deoxycytidine (5-dAzaC), Trichostatin A (TSA)

## Abstract

**Purpose:**

Recent studies indicated undisputed contribution of connective tissue growth factor (CTGF) in the development of many cancers, including non-small cell lung cancer (NSCLC). However, the functional role and regulation of *CTGF* expression during tumorigenesis remain elusive. Our goal was to determine CTGF transcript and protein levels in tumoral and matched control tissues from 98 NSCLC patients, to correlate the results with clinicopathological features and to investigate whether the *CTGF* expression can be epigenetically regulated in NSCLC.

**Methods:**

We used quantitative PCR, Western blotting and immunohistochemistry to evaluate *CTGF* expression in lung cancerous and histopathologically unchanged tissues. We tested the impact of 5-Aza-2′-deoxycytidine (5-dAzaC) and trichostatin A (TSA) on CTGF transcript and protein levels in NSCLC cells (A549, Calu-1). DNA methylation status of the *CTGF* regulatory region was evaluated by bisulfite sequencing. The influence of 5-dAzaC and TSA on NSCLC cells viability and proliferation was monitored by the trypan blue assay.

**Results:**

We found significantly decreased levels of CTGF mRNA and protein (both *p* < 0.0000001) in cancerous tissues of NSCLC patients. Down-regulation of *CTGF* occurred regardless of gender in all histological subtypes of NSCLC. Moreover, we showed that 5-dAzaC and TSA were able to restore CTGF mRNA and protein contents in NSCLC cells. However, no methylation within *CTGF* regulatory region was detected. Both compounds significantly reduced NSCLC cells proliferation.

**Conclusions:**

Decreased expression of *CTGF* is a common feature in NSCLC; however, it can be restored by the chromatin-modifying agents such as 5-dAzaC or TSA and consequently restrain cancer development.

**Electronic supplementary material:**

The online version of this article (doi:10.1007/s00432-016-2195-3) contains supplementary material, which is available to authorized users.

## Introduction

Lung cancer (LC) is the most common cancer in the world, with more than 1 million cases reported annually (Jemal et al. 2011). Due to its high mortality rate, it is also the leading cause of cancer-related death worldwide. Two major types of LC can be distinguished, basing on clinical classification: non-small cell lung cancer (NSCLC) and small cell lung cancer (SCLC). NSCLC is usually reported to comprise about 85 % of all lung carcinomas and includes the following histological subtypes: adenocarcinoma (ADC), squamous cell carcinoma (SCC), large cell carcinoma (LCC) and other rare types (Alì et al. 2008).

Although lung carcinogenesis is one of the most extensively studied disorders, it is a very complex process that still requires clarification. In these days, a lot of studies focus on the interplay between tumor cells and surrounding stromal cells with an extracellular matrix (ECM) that both create the tumor microenvironment (Mbeunkui and Johann 2009; Lu et al. 2012; Sainio and Järveläinen 2014). ECM is a complex network of macromolecules and secretory proteins that regulate cell–cell and cell–matrix interactions, cellular behavior, as well as tissue polarity and architecture. This network is commonly deregulated during malignant transformation and plays a crucial role in the tumor development and progression (Lu et al. 2012).

Connective tissue growth factor (CTGF) is a 38-kDa, cysteine-rich protein belonging to the CCN family (Lau and Lam 1999). Biologically active CTGF is widely expressed in various cells (e.g., fibroblasts, myofibroblasts, endothelial cells, smooth muscle cells and some epithelial cell types) and can be secreted to ECM or is located on the cell membrane (Chien et al. 2006; Jacobson and Cunningham 2012). Like other members of CCN family, CTGF possess multimodular structure which enables binding to and interacting with other molecules (Kubota and Takigawa 2015). Therefore, CTGF as a molecular mediator presents pleiotropic functions and plays a pivotal role in a wide variety of regulatory processes including ECM synthesis and rearrangement, wound healing, angiogenesis, cell adhesion, migration, proliferation and differentiation (Cicha and Goppelt-Struebe 2009; Ponticos et al. 2009).

So far, diverse studies have indicated variable properties of CTGF in different tissues, and its expression level is thought to be dependent on the cell type and context. A lot of cancers present the deregulated expression of *CTGF*, when compared with their normal counterparts, which favors tumor growth and progression (Jacobson and Cunningham 2012; Wells et al. 2014). However, the mechanism of CTGF action during carcinogenesis may depend on its basal level in normal, histopathologically unchanged tissue (Wells et al. 2014). An increased expression of *CTGF* was detected in multiple human cancers, e.g., in gliomas, papillary thyroid carcinomas, precursor B-cell acute lymphoblastic leukemias, hepatocellular carcinoma and malignant melanoma, and was associated with the development of those diseases (Braig et al. 2011; Edwards et al. 2011; Urtasun et al. 2011; Welch et al. 2013; Wang et al. 2013; Finger et al. 2014). On the contrary, this gene was shown to be down-regulated in lung and colon cancers and its diminished expression was correlated with poorer clinical outcome of patients (Lin et al. 2005; Chen et al. 2007a; Ladwa et al. 2011).

Few previous studies showed that the expression of *CTGF* can be epigenetically regulated (Kikuchi et al. 2007; Hemmatazad et al. 2009; Komorowsky et al. 2009; Welch et al. 2013). The most widely studied epigenetic changes in LC include DNA methylation within CpG dinucleotide-rich regions of various genes (CpG islands) and posttranslational modifications of histone tails that affect local chromatin architecture (Nelson et al. 2012; Balgkouranidou et al. 2013; Heller et al. 2013; Langevin et al. 2015). DNA methylation is conducted by DNA methyltransferases (DNMTs), and during carcinogenesis, it may lead to hypermethylation of the promoter regions of tumor suppressor genes, resulting in their transcriptional silencing, or to global hypomethylation that enhances protooncogene expression (Luczak and Jagodziński 2006). Histone acetylation and the opposite process, deacetylation, are mediated by two different sets of enzymes: histone acetyltransferases (HATs) and histone deacetylases (HDACs) that alter chromatin compaction and thus are involved in transcriptional regulation of gene expression (Nervi et al. 2015). To the best of our knowledge, there are no reports considering the impact of chemical compounds causing chromatin rearrangement on the expression level of *CTGF* in LC.

In the present study, we determined the status of CTGF in lung cancerous and corresponding histopathologically unchanged tissues obtained from 98 patients with NSCLC, at both mRNA and protein levels, and we correlated them with clinicopathological features. Next, we examined the effect of 5-Aza-2′-deoxycytidine (5-dAzaC), a well-known DNMTs inhibitor, and trichostatin A (TSA), a potent HDACs inhibitor, on the *CTGF* expression level in two NSCLC cell lines belonging to different histological subtypes—A549 (ADC) and Calu-1 (SCC). We also assessed the impact of those compounds on cell viability and proliferation.

## Materials and methods

### Antibodies and reagents

Goat polyclonal anti-CTGF antibody (Ab) (L-20), rabbit polyclonal anti-glyceraldehyde-3-phosphate (GAPDH) Ab (FL-335), rabbit anti-goat and goat anti-rabbit horseradish peroxidase (HRP)-conjugated Ab were purchased from Santa Cruz Biotechnology (Santa Cruz, CA). TRI Reagent^®^, 5-dAzaC, TSA, dimethyl sulfoxide (DMSO), ethanol, fetal bovine serum (FBS), cell culture antibiotics and media were provided by Sigma-Aldrich Co. (St. Louis, MO).

### Patient material

Primary lung cancerous and histopathologically unchanged lung tissues, located at least 10–20 cm away from the cancerous lesions, were obtained between March 2012 and December 2014 from 98 patients diagnosed with NSCLC, who underwent surgical resection at the Department of Thoracic Surgery, Poznan University of Medical Sciences, Poland (Tables 1, 2, 3; Supplementary tables 1 and 2). Among them, 18 patients were never smokers. None of the patients received any preoperative chemotherapy and/or radiation therapy. Histopathological classification was performed by an experienced pathologist. After surgical removal, tissue samples were immediately snap-frozen in liquid nitrogen and stored at −80 °C until further processing. All patients participated in this study had signed informed consent on the use of clinical specimens, and the study was approved by the Local Ethical Committee of Poznan University of Medical Sciences.

**Table 1:** Differences in *CTGF* transcript levels in lung cancerous and corresponding histopathologically unchanged tissues from NSCLC patients including clinicopathological characteristic

Variables	Number of cases	Cancerous tissues	Histopathologically unchanged tissues	*p* value *CTGF* mRNA
		Mean ± SD	Mean ± SD	
Total no. of patients	98	2.58 ± 0.41	3.12 ± 0.4	<0.0000001
Gender				
Male	63	2.60 ± 0.44	3.08 ± 0.40	<0.0000001
Female	35	2.54 ± 0.35	3.19 ± 0.38	<0.0000001
Patient age				
≤60 (males; females)	26 (17; 9)	2.61 ± 0.39	3.16 ± 0.31	<0.000001
>60	72 (46; 26)	2.57 ± 0.41	3.10 ± 0.42	<0.0000001
Histological type				
Adenocarcinoma (males; females)	39 (22; 17)	2.5 ± 0.33	3.04 ± 0.39	<0.0000001
Squamous cell carcinoma	48 (36; 12)	2.67 ± 0.44	3.11 ± 0.41	<0.000001
Large cell carcinoma	5 (3; 2)	2.54 ± 0.33	3.39 ± 0.13	0.0007
Carcinoid	6 (2; 4)	2.44 ± 0.60	3.46 ± 0.21	0.005
Lung cancer stages				
0 (males; females)	5 (3; 2)	2.45 ± 0.55	3.13 ± 0.50	0.08
1A	11 (5; 6)	2.70 ± 0.48	3.19 ± 3.33	0.01
1B	22 (10; 12)	2.52 ± 0.33	2.98 ± 0.34	0.00005
2A	24 (15; 9)	2.59 ± 0.34	3.17 ± 0.42	0.000004
2B	16 (13; 3)	2.63 ± 0.39	3.13 ± 0.5	0.003
3A	19 (16; 3)	2.53 ± 0.52	3.15 ± 0.37	0.0001
3B	–	–	–	–
4	1 (1; 0)	–	–	–

*CTGF* transcript levels were standardized by the geometric mean of *PBGD* and *hMRPL19* cDNA levels. Results were expressed as decimal logarithm of multiples of cDNA copies in the calibrator. The normality of observed patient data distribution was assessed by Shapiro–Wilk test, and parametric unpaired, two-tailed *t* test was used to compare the mean values. *p* < 0.05 was considered as statistically significant

**Table 2:** Differences in CTGF protein levels in lung cancerous and corresponding histopathologically unchanged tissues from NSCLC patients including clinicopathological characteristic

Variables	Number of cases	Cancerous tissues	Histopathologically unchanged tissues	*p* value CTGF protein
		Mean ± SD	Mean ± SD	
Total no. of patients	98	2.79 ± 0.32	3.06 ± 0.34	<0.0000001
Gender				
Male	63	2.77 ± 0.32	3.11 ± 0.32	<0.0000001
Female	35	2.8 ± 0.32	3.02 ± 0.39	0.02
Patient age				
≤60 (males; females)	26 (17; 9)	2.72 ± 0.35	2.85 ± 0.45	0.26
>60	72 (46; 26)	2.81 ± 0.31	3.16 ± 0.27	<0.0000001
Histological type				
Adenocarcinoma (males; females)	39 (22; 17)	2.72 ± 0.31	3.04 ± 0.43	0.0005
Squamous cell carcinoma	48 (36; 12)	2.78 ± 0.32	3.08 ± 0.3	0.00001
Large cell carcinoma	5 (3; 2)	2.89 ± 0.09	3.15 ± 0.16	0.01
Carcinoid	6 (2; 4)	3.21 ± 0.16	3.2 ± 0.3	0.9
Lung cancer stages				
0 (males; females)	5 (3; 2)	2.98 ± 0.36	3.18 ± 0.54	0.6
1A	11 (5; 6)	2.91 ± 0.32	3.24 ± 0.18	0.007
1B	22 (10; 12)	2.71 ± 0.29	3.09 ± 0.28	0.0002
2A	24 (15; 9)	2.70 ± 0.32	3.04 ± 0.39	0.002
2B	16 (13; 3)	2.77 ± 0.37	2.87 ± 0.39	0.5
3A	19 (16; 3)	2.84 ± 0.28	3.18 ± 0.32	0.003
3B	–	–	–	–
4	1 (1; 0)	–	–	–

The amount of proteins detected by Western blotting was presented as the decimal logarithm of CTGF-to-GAPDH band optical density ratio. The normality of observed patient data distribution was assessed by Shapiro–Wilk test, and parametric unpaired, two-tailed *t* test was used to compare the mean values. *p* < 0.05 was considered as statistically significant

**Table 3:** Association between CTGF transcript and protein levels in lung cancerous tissues and different clinicopathological parameters

Variables	Number of cases	CTGF mRNA		CTGF protein	
		Mean ± SD	*p*	Mean ± SD	*p*
Gender			0.41^§^		0.61^§^
Male	63	2.60 ± 0.44		2.77 ± 0.32	
Female	35	2.54 ± 0.35		2.80 ± 0.32	
Patient age			0.62^§^		0.73^§^
≤60	26	2.61 ± 0.39		2.72 ± 0.35	
>60	72	2.57 ± 0.41		2.81 ± 0.31	
Histologic type			0.13^#^		0.047^#^
Adenocarcinoma	39	2.49 ± 0.33		2.78 ± 0.29	
Squamous cell carcinoma	48	2.67 ± 0.44		2.83 ± 0.35	
Large cell carcinoma	5	2.43 ± 0.41		2.84 ± 0.32	
Carcinoid	6	2.47 ± 0.54		3.18 ± 0.25	0.026^a^
Lung cancer stages			0.85^#^		0.37^#^
0–1	38	2.57 ± 0.41		2.89 ± 0.34	
2	40	2.60 ± 0.36		2.79 ± 0.34	
3–4	20	2.52 ± 0.51		2.90 ± 0.29	
Tumor size			0.69^‡^		0.78^#^
Tis–T1	21	2.62 ± 0.47		2.87 ± 0.35	
T2	57	2.56 ± 0.33		2.81 ± 0.34	
T3–T4	20	2.60 ± 0.52		2.82 ± 0.29	
Lymph node metastasis			0.09^#^		0.83^#^
N0	54	2.56 ± 0.40		2.78 ± 0.33	
N1	34	2.67 ± 0.40		2.80 ± 0.33	
N2	10	2.35 ± 0.42		2.79 ± 0.26	
Distant metastasis			0.23^§^		0.58^§^
M0	95	2.57 ± 0.41		2.78 ± 0.32	
M1a	3	2.86 ± 0.43		2.80 ± 0.36	
Smoking			0.43^§^		0.86^§^
Yes	80	2.59 ± 0.42		2.83 ± 0.34	
No	18	2.51 ± 0.32		2.82 ± 0.27	

^**§**^ *t* test

^**#**^ANOVA

^a^Post hoc RIR Tukey’s test

^‡^Kruskal–Wallis test

### Cell culture

The human NSCLC cell lines—A549 and Calu-1, breast cancer cell line—T47D, and cervical carcinoma cell line—HeLa, were purchased from ATCC (Rockville, MD). Normal human bronchial epithelial cells—Beas-2B, were kindly provided by Dr M. Rusin from the Maria Skłodowska-Curie Memorial Cancer Center and Institute of Oncology, Gliwice Branch, Poland. A549, Calu-1 and T47D cells were routinely maintained in RPMI 1640 medium, Beas-2B cell line was cultured in DMEM/F12 medium, and HeLa cell line was maintained in DMEM. Cells were grown at 37 °C in humidified air with 5 % CO_2_. All culture media were supplemented with 10 % heat-inactivated FBS, 2 mM glutamine and penicillin–streptomycin–amphotericin B solution (10,000 U penicillin, 10 mg streptomycin and 25 μg amphotericin B/ml).

### NSCLC cell lines treatment with 5-dAzaC and TSA

The stock solutions of 5-dAzaC (10 mg/ml) and TSA (1 mg/ml) were prepared in DMSO and ethanol, respectively, aliquoted and stored at −20 °C until use. Prior to all experiments, cells were seeded and grown overnight. Next, the investigated compounds were diluted in the culture medium to the desired concentration and added to cell cultures. The same volume of DMSO or ethanol was used as a vehicle control, and their final concentration in culture medium never exceeded 0.1 %. All experimental media were exchanged every 24 h. To determine the effect of 5-dAzaC on *CTGF* transcript and protein levels in NSCLC cell lines, A549 and Calu-1 cells were cultured for 48, 72 and 96 h either in the absence or in the presence of 5-dAzaC at concentrations of 10 and 15 μM. In order to evaluate whether TSA may regulate *CTGF* transcript and protein contents in A549 and Calu-1 cell lines, cells were treated with different concentrations of TSA (30, 100, 300 nM) or vehicle control in a time-dependent manner for 12, 24, 48 and 72 h. Each experiment was performed in triplicate, and cells were used for protein and RNA isolation, Western blotting and RT-qPCR analysis. Additionally, genomic DNA was isolated from cells cultured in the absence or in the presence of 10 μM 5-dAzaC for 96 h in order to evaluate its impact on the methylation status of *CTGF* regulatory region.

### Assessment of A549 and Calu-1 cells viability and proliferation after 5-dAzaC and TSA treatment by a trypan blue staining

A549 and Calu-1 cells were seeded into T-25-cm^2^ flasks and treated with 5-dAzaC or TSA as described above. After each incubation period, cells were detached with Trypsin–EDTA solution, Sigma-Aldrich Co. (St. Louis, MO), centrifuged and resuspended in 8 ml of phosphate-buffered saline (PBS). Next, cell viability and proliferation were determined by a trypan blue staining. Fifty μl of cell suspension was taken and mixed with the same volume of trypan blue. Cells were counted with an EVE™ automatic cell counter, NanoEnTek Inc. (Seoul, Korea). For cell viability, results are expressed as the percentage of viable cells relative to respective controls (100 %), and for cell proliferation, the number of counted cells per 1 ml of solution is presented. All experiments were performed in triplicate, and data represent mean ± standard deviation (SD).

### Reverse transcription and real-time quantitative polymerase chain reaction (RT-qPCR) analysis

Total RNA from lung tumors and matched normal lung tissues obtained from the same patients, as well as from investigated cell lines, was isolated according to the method of Chomczyński and Sacchi (1987). RNA samples were quantified and reverse-transcribed into cDNA using M-MLV reverse transcriptase from Invitrogen, Life Technologies (Grand Island, NY), according to the manufacturer’s protocol. The RT-qPCR was conducted in a Light Cycler^®^480 Real-Time PCR System, Roche Diagnostics GmbH (Mannheim, Germany), using SYBR Green I as a detection dye. The target cDNA was quantified by relative quantification method using a calibrator for primary tissues or respective controls for A549 and Calu-1 cells. For the calibrator, 1 µl of cDNAs from all tissue samples was mixed together. The quantity of *CTGF* transcript in each sample was standardized by the geometric mean of *porphobilinogen deaminase* (*PBGD*) and *human mitochondrial ribosomal protein L19* (*hMRPL19*) cDNA levels. The PCR amplification efficiency for target and reference genes was determined by the different standard curves, created by consecutive dilutions of the cDNA template mixture, as provided in Relative Quantification Manual, Roche Diagnostics GmbH (Mannheim, Germany). For amplification, 1 µl of total (20 µl) cDNA solution was added to 9 µl of Light Cycler^®^480 SYBR Green I Master mix (1 × concentrated) containing 2.5 mM MgCl_2_ and 0.5 µM primers (Supplementary table 3). A sample of no reverse-transcribed RNA and a no-template control were included in each batch of samples to provide a negative control in subsequent PCR. Melting curve analysis and electrophoresis were applied to confirm the specificity of the amplified products. All experiments were performed in triplicate, and *CTGF* transcript levels in investigated tissues were expressed as the decimal logarithm of multiplicity of cDNA concentrations in the calibrator. *CTGF* transcript levels for A549 and Calu-1 cell lines treated with 5-dAzaC or TSA were presented as multiplicity of the respective controls.

### Sodium dodecyl sulfate–polyacrylamide gel electrophoresis (SDS–PAGE) and Western blotting analysis

Cells were harvested using trypsin–EDTA solution, washed twice with PBS and lysed using RIPA lysis buffer, Sigma-Aldrich Co. (St. Louis, MO). Tissue specimens were homogenized in liquid nitrogen and also treated with RIPA lysis buffer. In both cases, RIPA buffer was supplemented with protease inhibitor cocktail, Roche Diagnostics GmbH (Mannheim, Germany). Samples were incubated on ice for 30 min, with vortexing every 15 min, and then centrifuged at 10,000×*g* for 10 min at 4 °C in order to remove cell debris. Supernatant was collected for whole cell lysates. The concentration of total protein isolated from cell lines was determined by the bicinchoninic acid assay method (BCA) using BCA kit from Sigma-Aldrich Co. (St. Louis, MO). Next, proteins (30 µg for cell lines) were resuspended in a sample loading buffer, boiled at 95 °C for 10 min, rapidly cooled on ice and separated on 12 % Tris–glycine gel using SDS–PAGE. Gel proteins were transferred to a nitrocellulose membrane, which was then blocked with 5 % nonfat dry milk in 1× concentrated Tris-buffered saline/Tween 20 for 2 h at room temperature, on a shaker. After blocking, membranes were incubated overnight at 4 °C with goat polyclonal anti-CTGF Ab (L-20) at a dilution of 1:500, followed by 2-h incubation with rabbit anti-goat HRP-conjugated Ab (1:5000). To ensure protein loading control of the lanes, membranes were stripped and incubated with rabbit polyclonal anti-GAPDH Ab (FL-335; 1:3300), followed by incubation with goat anti-rabbit HRP-conjugated Ab (1:5000). Bands were revealed using SuperSignal West Femto Chemiluminescent Substrate, Thermo Fisher Scientific (Rockford, IL) and Biospectrum^®^ Imaging System 500, UVP Ltd. (Upland, CA). The amounts of analyzed proteins were presented as the CTGF-to-GAPDH band optical density ratio. For A549 and Calu-1 cells cultured in the absence of 5-dAzaC or TSA, the ratio of CTGF to GAPDH was assumed to be 1. Additionally, HeLa cell line lysate was used as a positive control for CTGF protein identification according to CTGF Ab datasheet.

### Immunohistochemistry

Formalin-fixed, paraffin wax-embedded tissue specimens were cut on 4-μm sections and mounted on adhesion microscope slides SuperFrost^®^Plus (Menzel Gläser). Next, sections were dewaxed and rehydrated, and heat-induced antigen retrieval was carried out by cooking in low pH EnVision FLEX Target Retrieval Solution, Dako (Glostrup, Denmark), for 50 min at 97 °C. Endogenous peroxidase was blocked by EnVision FLEX Peroxidase-Blocking Reagent, Dako (Glostrup, Denmark), and sections were incubated in Novocastra Protein Block, Leica Biosystems (Wetzlar, Germany), for 10 min followed by overnight incubation with goat polyclonal anti-CTGF Ab (L-20; dilution 1:200) in a humid chamber at 4 °C. The primary antibody was diluted in EnVision FLEX Antibody Diluent, Dako (Glostrup, Denmark). Immunodetection was achieved using the LSAB System, Dako (Glostrup, Denmark), which is a two-step streptavidin–biotin–HRP method. Each layer was incubated for half an hour at room temperature. Between each staining steps slides were washed in EnVision FLEX Wash Buffer, Dako (Glostrup, Denmark). Visualization was achieved using 3-3′-diaminobenzidine tetrachloride (DAB, Leica Microsystems). Sections were counterstained with Mayer’s hematoxylin, dehydrated, cleared and mounted in DPX. Sections from formalin-fixed and paraffin-embedded normal human lung were used as positive control. Moreover, the presence of CTGF staining in histopathologically unchanged respiratory epithelium, macrophages and stromal fibroblasts within the tumor sections served as an internal positive control. Immunohistochemical staining was evaluated by experienced pathologist.

### Evaluation of DNA methylation status of the *CTGF* CpG-rich region by bisulfite sequencing and by MS-HRM analysis

The position of CpG islands in the *CTGF* regulatory region was determined by online programs (UCSC Genome Bioinformatics Site and EMBOSS CpGPlot/CpGReport/Isochore). Methylation status of 106 CpG islands located at Chr6: 132271726–132272591 (according to UCSC GRCh37/hg19) was evaluated by sequencing of bisulfite-modified DNA fragment amplified by following primers (Supplementary table 3). Genomic DNA, from A549 and Calu-1 cells, cultured in the absence or in the presence of 10 μM 5-dAzaC for 96 h and from lung cancerous and histopathologically unchanged tissues of 98 NSCLC patients, was isolated using DNA Mammalian Genomic Purification Kit, Sigma-Aldrich Co. (St. Louis, MO). Next, 500 ng of genomic DNA was subjected to bisulfite conversion according to EZ DNA Methylation Kit™ protocol, Zymo Research Corporation **(**Orange, CA).

Methylation-sensitive high-resolution melting analysis (MS-HRM) was used as a screening method for the detection if there are any differences in DNA methylation patterns between lung cancerous and histopathologically unchanged tissues from NSCLC patients. Methylation level of DNA fragment located within the CpG island of *CTGF* gene was determined by RT-PCR amplification of bisulfite-modified DNA, followed by HRM profile analysis. The reaction was conducted in a Light Cycler^®^480 Real-Time PCR System, Roche Diagnostics GmbH (Mannheim, Germany). The *CTGF* region containing 15 CpG dinucleotides located at Chr6: 132271312–132271581 (according to UCSC GRCh37/hg19) was amplified by a pair of primers complementary to the bisulfite-DNA-modified sequence (Supplementary table 3). For PCR amplification, 1 μl of the bisulfite-treated DNA from patients was added to 10 μl of 5× HOT FIREPol^®^ EvaGreen^®^ HRM Mix, Solis BioDyne Co. (Tartu, Estonia) with 0.2 μM primers. Each reaction was performed in triplicate. MS-HRM analysis was performed using Light Cycler^®^480 Gene Scanning software and TM Calling software. The methylated and unmethylated DNA acquires different sequences after bisulfite treatment resulting in different melting profiles of PCR products. We analyzed the shape of achieved melting curves among lung cancerous and histopathologically unchanged tissues, and when differences were detected, samples were targeted for bisulfite sequencing.

For bisulfite sequencing, bisulfite-modified DNA fragment was amplified with FastStart Taq DNA Polymerase from Roche Diagnostics GmbH (Mannheim, Germany). The PCR products were separated on agarose gel, purified using Agarose Gel DNA Extraction Kit, Roche (Mannheim, Germany), and cloned into pGEM-T Easy Vector System I, Promega (Madison, WI). After overnight ligation, competent TOPO10 *E. coli* strain cells were transformed with plasmids. Plasmid DNA isolated from five positive bacterial clones was used for commercial sequencing. The results of bisulfite sequencing were presented using BiQ analyzer software and the Bisulfite sequencing Data Presentation and Compilation (BDPC) web server, respectively (Bock et al. 2005; Rohde et al. 2009).

### Statistical analysis

The normality of the observed patient data distribution was assessed by Shapiro–Wilk test. Parametric unpaired, two-tailed *t*-test was used to consider statistically significant differences of CTGF mRNA and protein levels between lung cancerous and histopathologically unchanged tissues (*p* < 0.05). Multivariate regression was performed to detect association between histological type of cancer, smoking history and CTGF mRNA and protein levels in cancerous tissues. Parametric unpaired, two-tailed *t*-test and ANOVA with post hoc RIR Tukey’s test were used to compare normally distributed variables between groups. Otherwise, Kruskal–Wallis test was performed. Data groups for cell lines were assessed by ANOVA to evaluate whether there was significance (*p* < 0.05) between the groups. For all experimental groups, which fulfilled the initial criteria, individual comparisons were made by post hoc Tukey’s test with the assumption of two-tailed distribution. Data are expressed as the mean ± SD. Statistical analysis was performed with STATISTICA 12 software.

## Results

### *CTGF* transcript and protein levels are significantly decreased in lung cancerous tissues and are correlated with various clinicopathological features of NSCLC patients

**Fig. 1:**
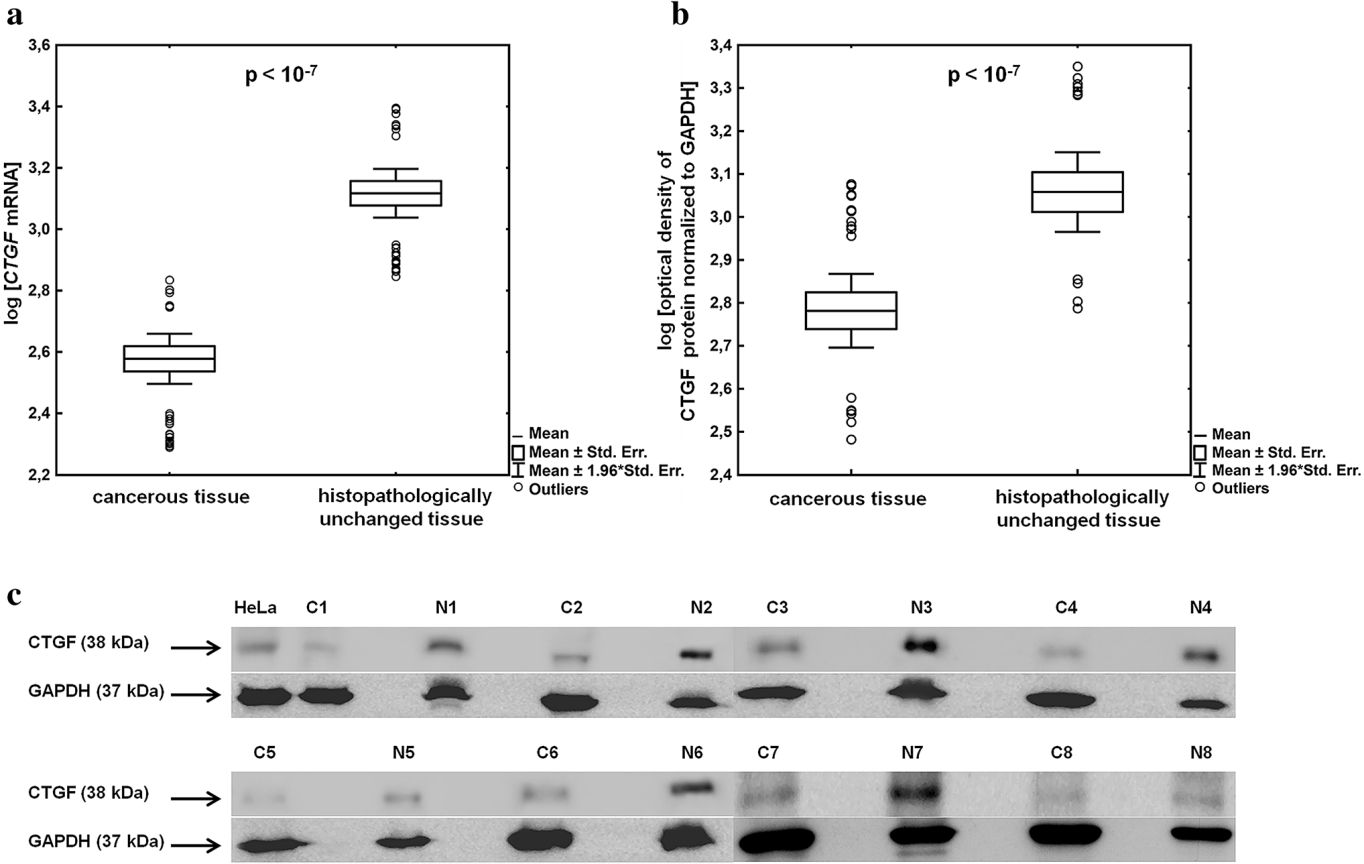
CTGF transcript and protein levels in lung cancerous and histopathologically unchanged tissues obtained from 98 patients with NSCLC. **a** *CTGF* transcript levels in NSCLC patients’ tissues. Total RNA was reverse-transcribed and cDNAs were studied by RT-qPCR analysis. Target cDNA was quantified by relative quantification method using a calibrator. The amount of *CTGF* mRNA was expressed as the decimal logarithm of multiples of cDNA copies in the calibrator. **b** CTGF protein levels in NSCLC patients’ tissues. Isolated proteins were separated by 12 % SDS-PAGE and detected by Western blotting. The band densitometry readings were normalized to GAPDH loading control. The amount of CTGF proteins detected in tissues was presented as the decimal logarithm of CTGF-to-GAPDH band optical density ratio. **c** Representative picture of Western blot analysis in lung cancerous tissues (C) and their matched histopathologically unchanged tissues (N) obtained from patients with NSCLC. HeLa cell line lysate was used as a positive control for CTGF protein identification. The *p* value in each graph was evaluated by parametric unpaired, two-tailed *t* test

In the present study, employing RT-qPCR and Western blotting, we found significantly lowered levels of CTGF transcript (*p* < 0.0000001) and protein (*p* < 0.0000001) in lung cancerous tissues compared with adjacent histopathologically unchanged tissues obtained from 98 patients with NSCLC (Fig. 1a–c). Of the 98 pairs, only 9 pairs (~9 %) presented higher amount of *CTGF* mRNA level in tumor tissue, 13 (~13 %) pairs presented higher CTGF protein content in cancerous tissue, and in 6 pairs (~6 %), *CTGF* mRNA level did not correspond to the protein level. To clarify the clinical significance of *CTGF* expression in NSCLC, we showed the differences in CTGF mRNA and protein levels between lung cancerous and histopathologically unchanged tissues including various clinicopathological features (Tables 1, 2; Supplementary tables 1 and 2). A substantial decrease in *CTGF* mRNA levels was detected in lung cancerous tissues, regardless of gender (males and females: *p* < 0.0000001), patients age (age ≤ 60 years: *p* < 0.000001; age > 60 years: *p* < 0.0000001) and histological type of NSCLC (ADC: *p* < 0.0000001; SCC: *p* < 0.000001; LCC: *p* = 0.0007; carcinoid: *p* = 0.005; Table 1). Those differences were statistically significant in all LC stages except stage 0 (*p* = 0.08). As shown in supplementary table 1, lowered *CTGF* transcript levels in lung cancerous tissues compared with matched normal specimens were associated with almost all tumor sizes, with the exception of Tis and T4 and with all grades of lymph node metastasis. Western blotting results showed that significantly lower amount of CTGF protein in lung cancerous tissues was detected at both genders (males: *p* < 0.0000001; females: *p* = 0.02) and in ADC (*p* = 0.005), SCC (*p* = 0.00001) and LCC (*p* = 0.01) histological subtypes of NSCLC (Table 2). Moreover, the decreased amount of CTGF protein was associated with T1b (*p* = 0.02), T2a (*p* = 0.000002) and T3 (*p* = 0.0007) tumor size and with all grades of lymph node metastasis (N0: *p* = 0.000004; N1: *p* = 0.01; N2: *p* = 0.03; Supplementary table 2). However, the difference in CTGF protein level between cancerous and histopathologically unchanged tissues did not reach statistical significance in the group of patients under 60 years of age, in patients with recognized carcinoid and with LC stage 0 and 2B (Table 2). We cannot consider the association of *CTGF* expression level with distant metastasis as 95 patients presented no distant metastasis (M0). We performed multivariate analysis to investigate the association of CTGF mRNA and protein levels with a history of smoking and histological type of LC. All models were weak, with an adjusted *R*^2^ < 0.7. We did not find a significant association of *CTGF* expression level with smoking status. However, we observed that in the group of cancerous tissues CTGF protein level was associated with histological type of LC (*p* = 0.013). The highest amount of CTGF protein was detected in carcinoid tumors and the lowest amount in ADCs. We also analyzed the relationship between CTGF transcript and protein levels in cancerous tissues and different clinicopathological parameters (Table 3). We found that there was significant difference in CTGF protein levels among histological types of LC (*p* = 0.047). The amount of CTGF protein was significantly decreased in ADCs compared with carcinoid tumors (*p* = 0.026). We did not identify any statistically significant difference between CTGF transcript and protein levels and other clinicopathological variables such age, sex, LC stage, tumor size, lymph node metastasis or smoking (Table 3).

**Fig. 2:**
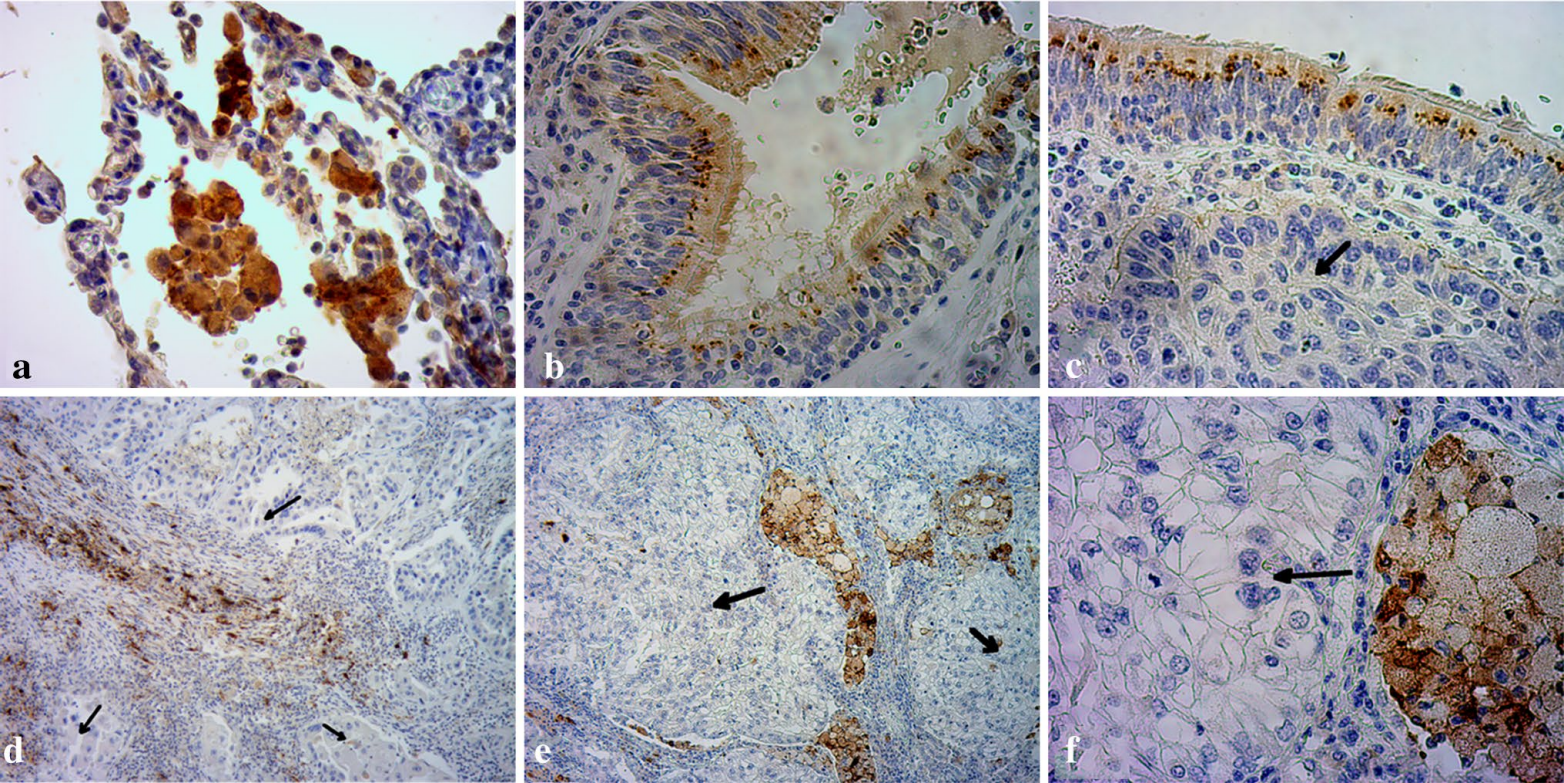
Representative immunohistochemical staining of CTGF in formalin-fixed, paraffin-embedded clinical tissue specimens. Positive immunoreactivity is indicated by the brown staining. Normal lung tissues were used as a positive control. **a** Normal lung tissue with strong CTGF staining detected in macrophages and some alveolar epithelial cells. **b** Moderate CTGF staining in normal respiratory epithelium (in normal epithelial cells lining the bronchial airway). **c**, **d** Representative immunohistochemistry images of SCC tissue specimens. SCC cells showing weak (**c**) or negative (**d**) staining for CTGF are indicated by *black arrows*. The presence of CTGF staining in normal respiratory epithelium (**c**) and in stromal fibroblasts (**d**) located within SCC tissue specimens served as an internal positive control. **e**, **f** Representative immunohistochemistry images of ADC specimens with indicated ADC cells showing no staining for CTGF (*black arrows*). Positive reaction was restricted to tumor-infiltrating macrophages (**e**, **f**). Original magnifications ×400 (**a**, **b**, **c**, **f**) and ×100 (**d**, **e**)

In order to complement our Western blotting results and to define the distribution and localization of CTGF protein in investigated patients’ material, representative NSCLC and non-tumoral lung specimens were immunohistochemically stained with anti-CTGF Ab (Fig. 2). Positive CTGF staining was found in normal lung tissues, with strong immunoreactivity in macrophages and in respiratory epithelium (Fig. 2a, b). SCC cells showed no or very weak cytoplasmic staining (Fig. 2c, d), whereas ADC cells were all negative for CTGF staining (Fig. 2e, f). However, the presence of CTGF protein was detected in tumor-infiltrating macrophages, stromal fibroblasts and normal bronchial epithelial cells located in the tumor field and was used as an internal positive control (Fig. 2c–f). Representative hematoxylin- and eosin-stained slides are shown in Supplementary figure 1.

Taken together, these results indicate that *CTGF* expression is significantly diminished in lung cancerous tissues of NSCLC patients; however, its exact role during tumorigenesis needs to be further investigated.

### Comparison of the basal *CTGF* expression level in NSCLC cells (A549, Calu-1), Beas-2B and T47D cell lines

**Fig. 3:**
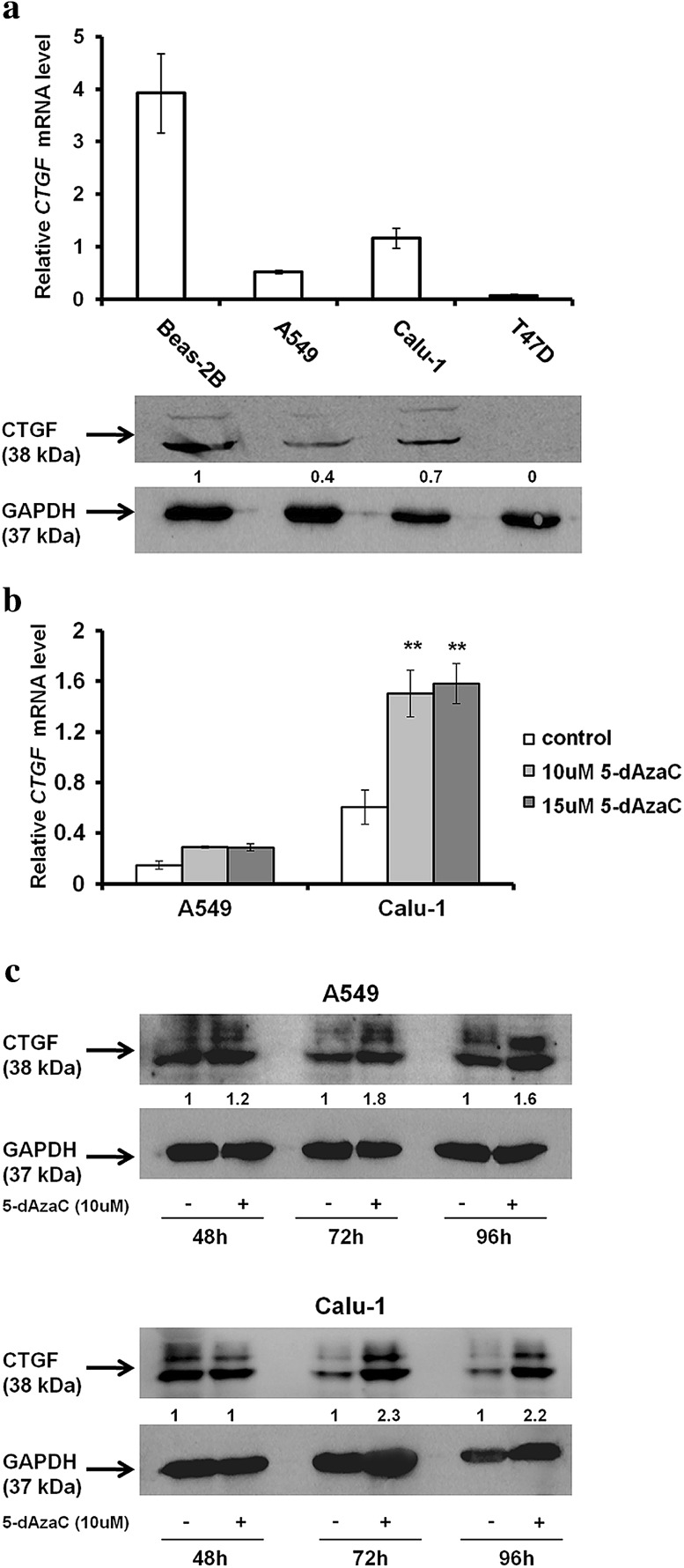
Basal and 5-dAzaC-induced expression of *CTGF* in NSCLC cell lines. **a** Comparison of the CTGF mRNA and protein abundance between NSCLC cells (A549, Calu-1), Beas-2B and T47D cells. Cells were cultured for 72 h in an appropriate medium and then were used for total RNA and protein isolation. *CTGF* cDNA levels were determined by RT-qPCR. Each sample was determined in triplicate, and results represent mean ± SD from three experiments. Isolated proteins were separated by 12 % SDS-PAGE and detected by Western blotting. The band densitometry readings were normalized to GAPDH loading control. The ratio of CTGF to GAPDH protein for Beas-2B cells was assumed to be 1. **b** 5-dAzaC increases *CTGF* transcript levels in A549 and Calu-1 cells. Cells were cultured for 96 h in the absence or in the presence of 5-dAzaC (10 and 15 μM). Next, cells were used for total RNA isolation and RT-qPCR analysis. *CTGF* transcript levels are presented as multiplicity of the respective controls. Each sample was determined in triplicate and results represent mean ± SD from three experiments (**p < 0.001). **c** 5-dAzaC increases CTGF protein level in A549 and Calu-1 cells. Cells were cultured for 48, 72 and 96 h in the absence or in the presence of 5-dAzaC (10 μM). Next, 30 μg of protein isolated from each sample was separated by 12 % SDS-PAGE and detected by Western blotting. The band densitometry readings were normalized to GAPDH loading control. The ratio of CTGF to GAPDH protein for cells incubated in the absence of 5-dAzaC for each period of time was assumed to be 1

Basal expression patterns of *CTGF* in investigated cells were determined by RT-qPCR, and the amount of CTGF protein was detected by Western blotting, respectively (Fig. 3a). A549 and Calu-1 cells presented definitely lowered content of *CTGF* mRNA in comparison with Beas-2B cell line. We observed that *CTGF* mRNA levels were about 7.5 times lower in A549 cells and 3.4 times lower in Calu-1 cells than in Beas-2B cells. The amounts of CTGF protein were consistent with the RT-qPCR results (Fig. 3a). However, the content of CTGF transcripts and protein in NSCLC cells was higher than in T47D, which is known to express *CTGF* at very low levels (Chen et al. 2007b). CTGF mRNA and protein levels detected in A549 cells were reduced more than twofold in comparison with those from Calu-1 cells. As expected, these results confirmed that the expression level of *CTGF* in NSCLC cells is diminished.

### 5-dAzaC increases *CTGF* transcript and protein levels in A549 and Calu-1 cells

In order to determine whether the expression of *CTGF* can be epigenetically regulated in NSCLC cells, we investigated whether DNMTs inhibition by 5-dAzaC would exert any effect on the *CTGF* mRNA level. After 96 h of incubation with 5-dAzaC at concentrations of 10 and 15 μM, we observed an elevation of *CTGF* transcript levels in A549 and Calu-1 cells (Fig. 3b). However, this induction was statistically significant only for Calu-1 cell line (*p* < 0.001). We noticed an approximately twofold increase in *CTGF* mRNA levels in A549 cells and 2.5-fold increase in Calu-1 cells. The effect was not dose dependent as both concentrations of 5-dAzaC up-regulated *CTGF* expression to the similar level in each cell line. Next, we examined whether RT-qPCR results would correspond to CTGF protein content. The densitometric analysis of Western blotting bands revealed that 5-dAzaC (10 μM) was able to raise the CTGF protein amount in investigated cells after 72 and 96 h of incubation as compared to respective controls (Fig. 3c). The double bands (36 and 38 kDa) of full length, intact CTGF were detected (Zarrinkalam et al. 2003), and both were markedly increased after 5-dAzaC treatment. Moreover, we investigated whether the same effect, exerted by 5-dAzaC, would be observed in Beas-2B cells. We revealed that 5-dAzaC (10 uM) was also able to elevate CTGF mRNA and protein levels in Beas-2B cell line after 48, 72 and 96 h of treatment (Supplementary figure 2).

### 5-dAzaC significantly inhibits NSCLC cells proliferation without marked reduction in cell viability

**Fig. 4:**
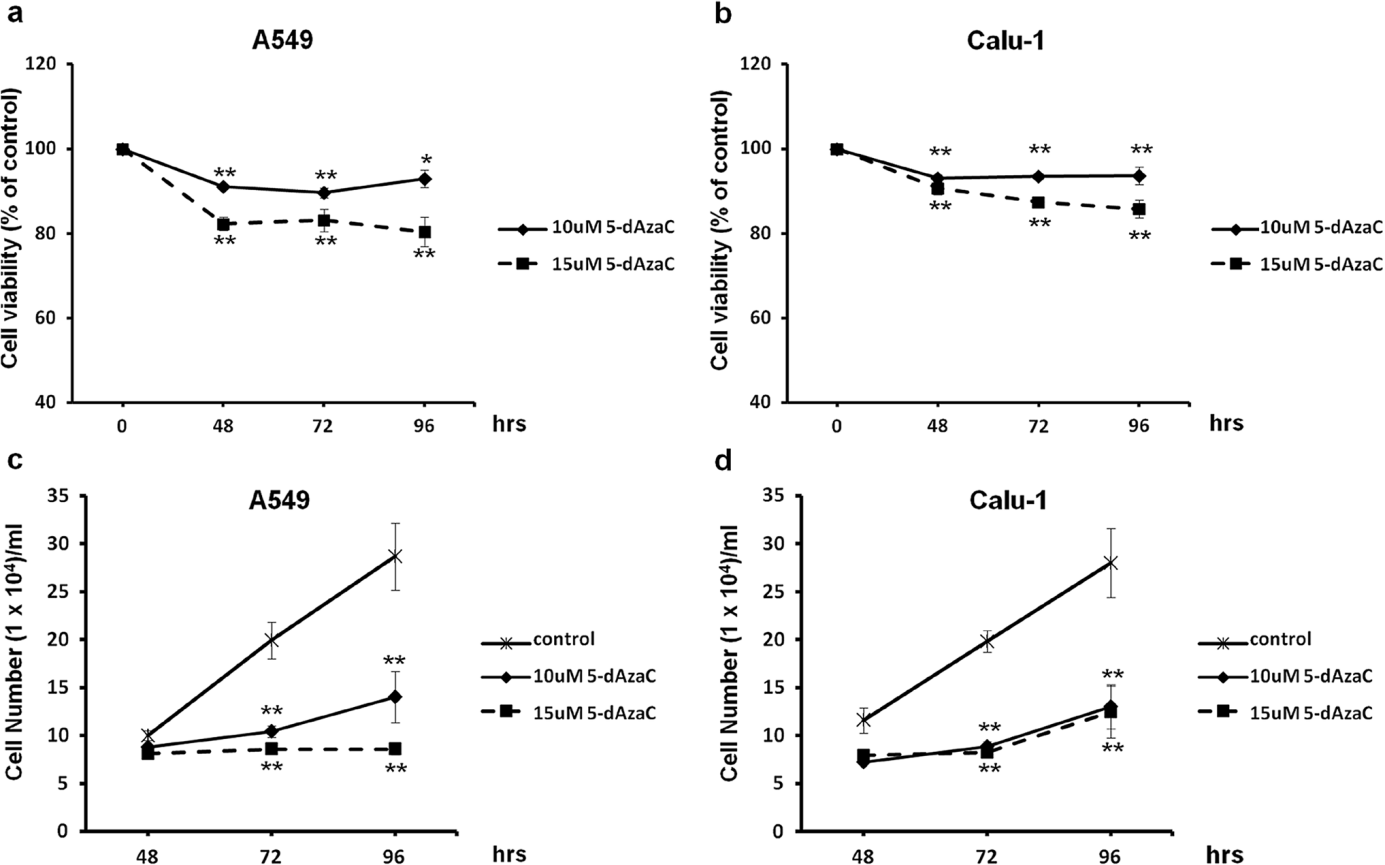
Effect of 5-dAzaC treatment on NSCLC cells viability and proliferation. Dose–response and time–course analysis of A549 and Calu-1 cell viability (**a**, **b**) and proliferation (**c**, **d**) after 5-dAzaC treatment. Cells were incubated in medium either in the absence or in the presence of 5-dAzaC at concentrations of 10 and 15 μM for 48, 72 and 96 h. After incubation, cell viability and proliferation were determined by a trypan blue exclusion assay. Cell viability results are expressed as a percentage of viable cells compared with respective untreated controls (100 %). For cell proliferation, the number of counted cells/ml is presented. All experiments were performed in triplicate, and results represent mean ± SD. Statistical significance: *p < 0.05; **p < 0.001 versus corresponding control

To determine whether concentrations of 5-dAzaC used in previous experiments do not exert a pronounced cytotoxic effect in investigated cell lines, we applied trypan blue exclusion assay for the measurement of cell viability. We showed that 5-dAzaC at concentrations of 10 and 15 µM reduced cell viability in a dose-dependent manner in both A549 and Calu-1 cell lines. The results were statistically significant at each time point (Fig. 4a, b). However, after 48, 72 and 96 h of incubation with 5-dAzaC (10 µM), A549 and Calu-1 cell viability was higher than 90 % compared with respective controls. The second dose of 5-dAzaC (15 µM) was more effective in reducing cell viability, although the amount of viable A549 and Calu-1 cells was still not lower than 80 %. On the contrary, both concentrations of 5-dAzaC were extremely potent in reducing the proliferation rate of A549 and Calu-1 cells. The cell growth inhibition was statistically significant and occurred after 72 h of incubation, for both cell lines, at both concentrations of 5-dAzaC (*p* < 0.001; Fig. 4c, d). Cell number counting by trypan blue assay revealed that after 96 h, 5-dAzaC (10 and 15 µM) reduced the proliferation of A549 cells by approximately 51 and 70 %, respectively (Fig. 4c). Both concentrations of investigated compound exhibited the same effect in Calu-1 cells at indicated time point, diminishing the proliferation rate by 54 % compared with untreated controls (Fig. 4d).

### DNA methylation status of *CTGF* regulatory region in A549, Calu-1 and Beas-2B cell lines and in lung cancerous and histopathologically unchanged tissues from patients with NSCLC

**Fig. 5:**
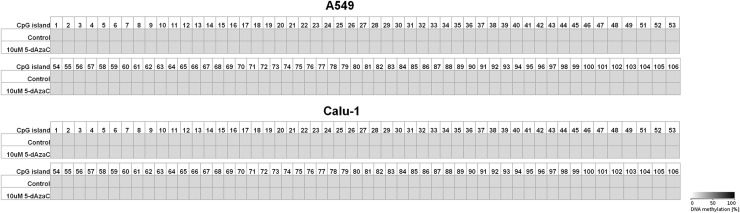
DNA methylation assessment of *CTGF* regulatory region by bisulfite sequencing in A549 and Calu-1 cell lines. A549 and Calu-1 cancer cells were cultured for 96 h either in the absence or in the presence of 5-dAzaC at a concentration of 10 μM. Then, cells were used for genomic DNA isolation, followed by bisulfite conversion of cytosine to uracil. The CpG-rich region containing 106 CpG dinucleotides was then amplified by a pair of primers complementary to the bisulfite-DNA-modified sequence (Supplementary table 3). The PCR products were purified with subsequent cloning into a plasmid vector. Plasmid DNA isolated from five bacterial clones was used for commercial sequencing. The results of bisulfite sequencing were assessed and presented using BiQ analyzer software and BDPC web server. Gray boxes represent unmethylated CpG dinucleotides

**Fig. 6:**
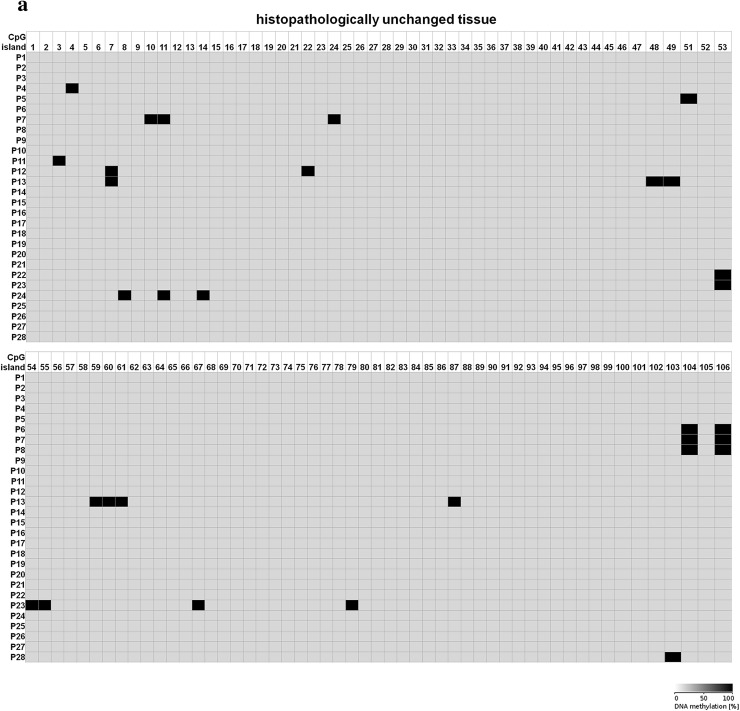

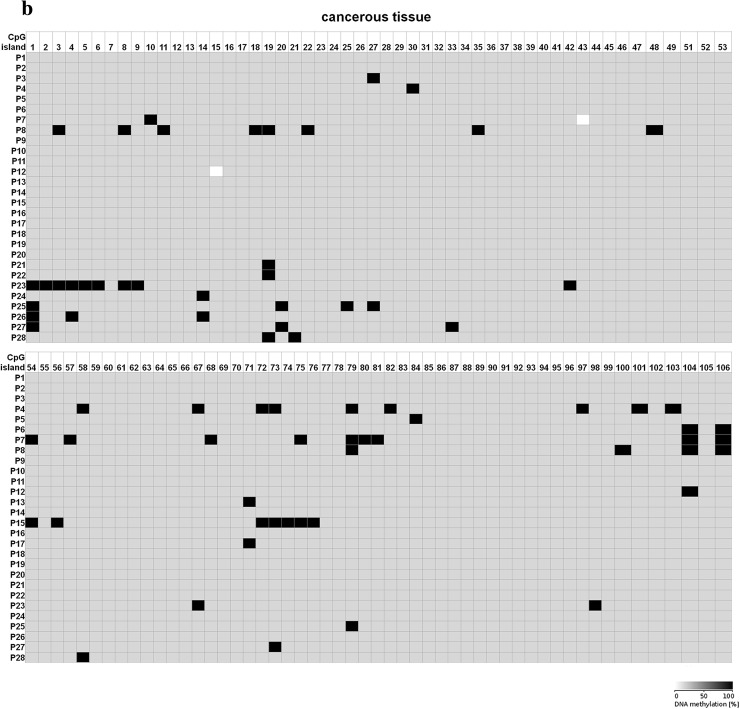
DNA methylation assessment of *CTGF* regulatory region by bisulfite sequencing in tissue samples obtained from 28 NSCLC patients. **a** Histopathologically unchanged tissues and **b** lung cancerous tissues obtained from 28 NSCLC patients were used for genomic DNA isolation, followed by its bisulfite conversion. **a**, **b** The CpG-rich region containing 106 CpG dinucleotides was then amplified by a pair of primers complementary to the bisulfite-DNA-modified sequence (Supplementary table 3). The PCR products were purified with subsequent cloning into a plasmid vector. Plasmid DNA isolated from five bacterial clones was used for commercial sequencing. The results of bisulfite sequencing were assessed and presented using BiQ analyzer software and BDPC web server. *Black* and *gray boxes* represent methylated and unmethylated CpG dinucleotide, respectively. *White boxes* correspond to undetermined CpG dinucleotides

Because 5-dAzaC was able to up-regulate *CTGF* mRNA and protein levels in A549, Calu-1 and Beas-2B cells, we decided to investigate whether those changes were caused by direct demethylation of the *CTGF* CpG-rich regulatory region. For that purpose, we incubated cells either in the absence or in the presence of 5-dAzaC (10 µM) for 96 h and then we examined the methylation status of 106 CpG islands within *CTGF* regulatory region (Chr6: 132271726–132272591) by bisulfite sequencing. Surprisingly, for both A549 and Calu-1 cells, no methylation in investigated region was detected (Fig. 5). The same result was obtained for Beas-2B cell line (Supplementary figure 3). These data suggest that 5-dAzaC is able to restore *CTGF* expression in NSCLC cells and in normal bronchial epithelial cells; however, it does not act through direct demethylation of *CTGF* regulatory region. We also evaluated the DNA methylation status of *CTGF* in lung cancerous and in matched normal tissues from 28 patients with NSCLC. We used MS-HRM analysis as a screening method in order to detect whether there are any differences in methylation status in *CTGF* regulatory region between lung cancerous and matched normal tissues in the group of 98 patients. We found 56 matched samples with different melting profiles of PCR products (Supplementary figure 4), and we further analyzed them by bisulfite sequencing. We detected different patterns of DNA methylation in examined region (Chr6: 132271726–132272591) between histopathologically unchanged tissues and lung cancerous tissues in the group of 28 NSCLC patients (Fig. 6a, b). Among those patients, 16 had SCC, 9 had ADC, 2 had LCC and 1 was diagnosed with carcinoid tumor. Changes in DNA methylation patterns between normal and cancerous tissues were not statistically significant (*p* = 0.08); however, this result is preliminary and further studies, on the larger group of patients, are needed to estimate whether DNA methylation plays any role in *CTGF* silencing in NSCLC.

### The effect of TSA on A549 and Calu-1 cell viability and proliferation

**Fig. 7:**
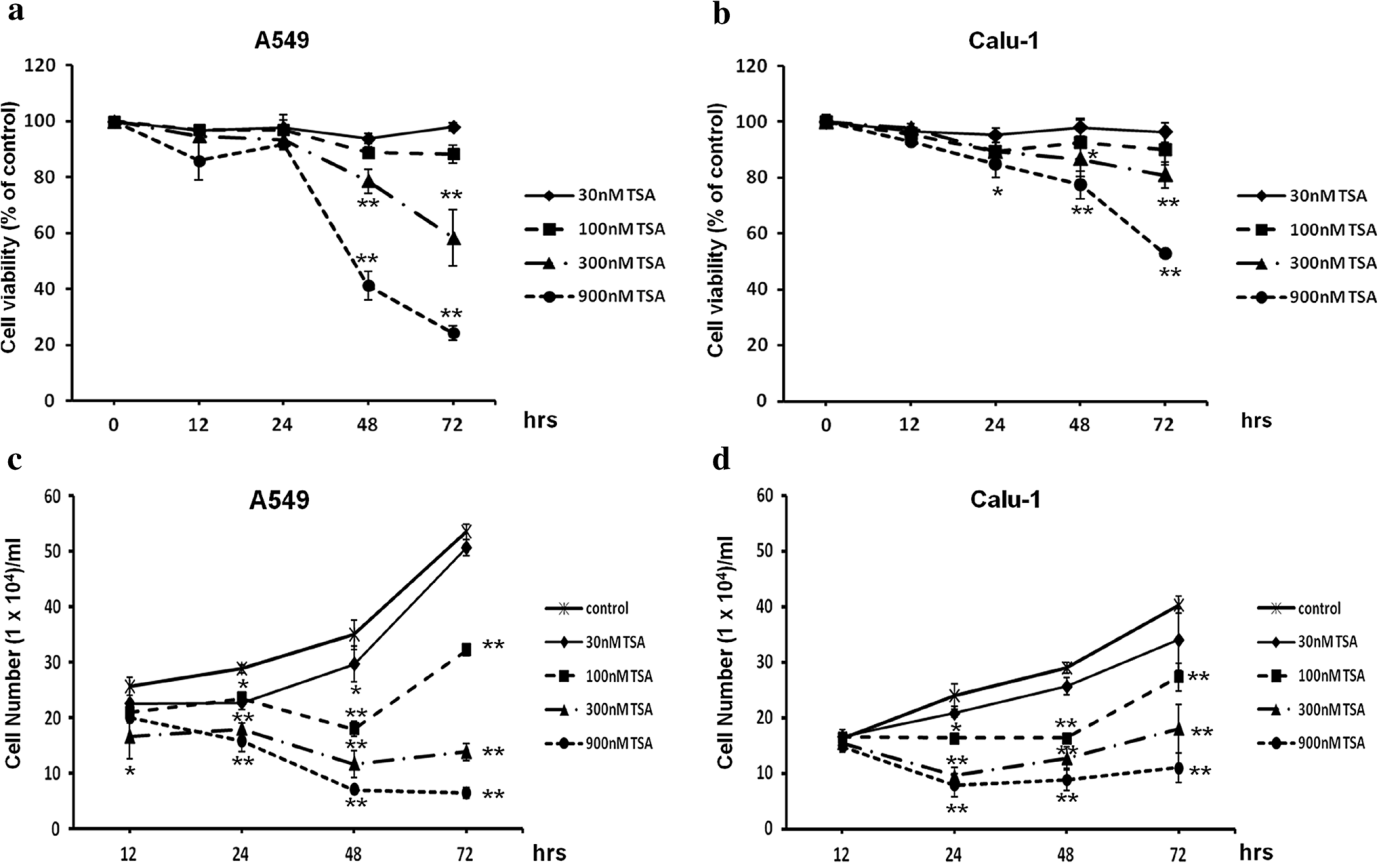
Effect of TSA treatment on NSCLC cells viability and proliferation. Dose–response and time–course analysis of A549 and Calu-1 cell viability (**a**, **b**) and proliferation (**c**, **d**) after TSA treatment. Cells were incubated for 12, 24, 48 and 72 h either in the absence or in the presence of TSA at concentrations of 30, 100, 300 and 900 nM. After incubation, cell viability and proliferation were determined by a trypan blue exclusion assay. Cell viability results were expressed as a percentage of viable cells compared to respective untreated controls (100 %). For cell proliferation, the number of counted cells/ml was presented. All experiments were performed in triplicate, and results represent mean ± SD. Statistical significance: *p < 0.05; **p < 0.001 versus corresponding control

Next, we investigated whether other epigenetic mechanism—histone acetylation, could restore the expression of *CTGF* in NSCLC cell lines. We chose one of the most potent HDACs inhibitor—TSA, and tested its impact on A549 and Calu-1 cell viability and proliferation, in order to estimate appropriate concentrations for further experiments. Different doses of TSA (30, 100, 300 and 900 nM) were applied to cell cultures for 12, 24, 48 and 72 h, and cell viability was evaluated by trypan blue staining and compared with ethanol-treated controls. Treatment with 900 nM of TSA markedly decreased A549 cell viability (41 % of controls after 48 h and 24 % of controls after 72 h) as well as Calu-1 cell viability (77 % of controls after 48 h and 53 % of controls after 72 h), and this concentration was excluded from further analysis (Fig. 7a, b). Moreover, we observed that A549 cell line is more vulnerable to TSA as significant cell morphology changes were detected after exposure to 900 nM of this compound. Other doses of TSA did not reduce cell viability to such extent and were used in other experiments. After 72 h of incubation with 300 nM of TSA, A549 cell viability was higher than 55 % and Calu-1 cell viability was higher than 80 % (Fig. 7a, b). Moreover, TSA appeared to be a highly potent inhibitor of NSCLC cells proliferation. The growth rate of A549 and Calu-1 cells after TSA treatment was significantly diminished in a dose and time-dependent manner compared with respective controls (Fig. 7c, d).

### TSA increases *CTGF* transcript and protein levels in A549 and Calu-1 cells

**Fig. 8:**
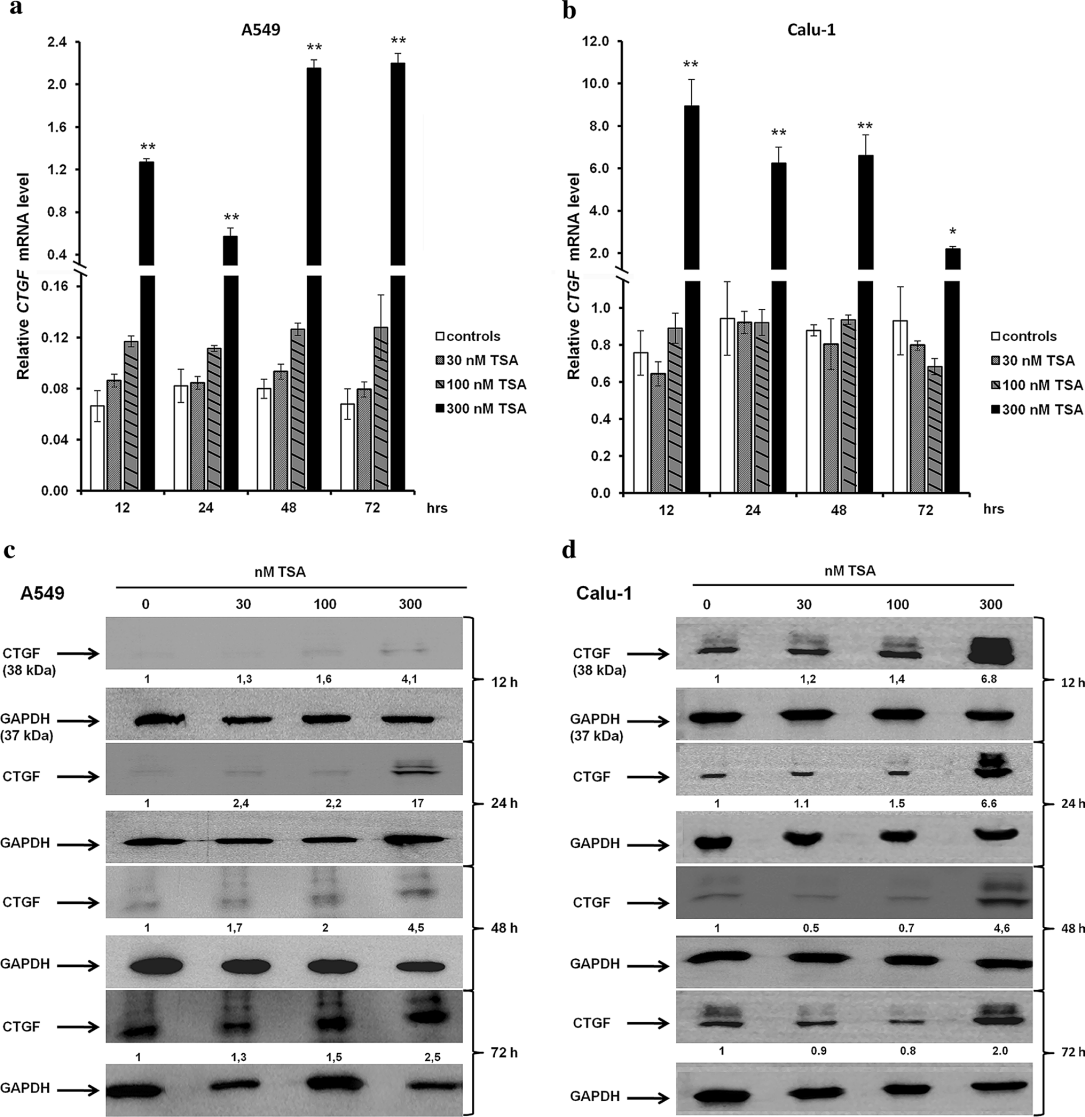
TSA increases CTGF transcript and protein contents in NSCLC cell lines. **a**, **b** An increment of *CTGF* transcript levels in A549 and Calu-1 cells after TSA treatment. Cells were cultured from 12 to 72 h either in the absence or in the presence of TSA at concentrations of 30, 100 and 300 nM. Next, cells were used for total RNA isolation and RT-qPCR analysis. *CTGF* transcript levels were presented as multiplicity of the respective controls. Each sample was determined in triplicate, and results represent mean ± SD from three experiments. Statistical significance: *p < 0.05; **p < 0.001 versus corresponding control. **c**, **d** TSA increases CTGF protein levels in A549 and Calu-1 cells. Cells were cultured from 12 to 72 h either in the absence or in the presence of TSA at concentrations of 30, 100 and 300 nM. Next, 30 μg of protein isolated from each sample was separated by 12 % SDS-PAGE and detected by Western blotting. The band densitometry readings were normalized to GAPDH loading control. The ratio of CTGF to GAPDH protein for cells incubated in the absence of TSA for each period of time was assumed to be 1

 We showed that TSA is able to regulate the expression of *CTGF* in A549 and Calu-1 cells. After treatment with increasing doses of TSA, we observed a progressive elevation of *CTGF* transcript levels in A549 cell line at each time point (Fig. 8a). Although the upward trend was visible for each concentration of TSA, statistically significant differences in *CTGF* mRNA levels between A549 treated and control cells were detected at 300 nM of TSA (*p* < 0.001; Fig. 8a). On the contrary, in Calu-1 cells only the highest tested dose of TSA (300 nM) resulted in an increase in *CTGF* transcript levels at each time of incubation (*p* < 0.001; Fig. 8b). Changes in *CTGF* transcript levels, induced by TSA, in both NSCLC cell lines were correlated with an elevation of CTGF protein amount (Fig. 7c, d). The densitometric analysis of Western blotting bands showed a progressive increase in CTGF protein levels after TSA treatment in A549 cells, in a dose-dependent manner at 12, 24, 48 and 72 h of incubation (Fig. 8c). The CTGF protein content in Calu-1 cells incubated with 300 nM of TSA was significantly elevated as compared to the respective controls at each time of incubation (Fig. 8d). Taken together, we showed that although A549 and Calu-1 cells vary in the sensitivity to TSA, this compound is able to restore the expression level of *CTGF* in those cell lines.

## Discussion

There are a lot of contradictory data describing the role of CTGF during carcinogenesis. However, the differences in CTGF action seem to be dependent on its basal expression level in original, non-tumoral cells. It has been reported that this growth factor enhances tumor development, when it is over-expressed in cancer cells compared with adjacent normal cells, or that it inhibits the proliferation and metastasis, when its expression pattern is restored in low-expressing cancer cells compared with non-transformed counterparts (Wells et al. 2014). Besides, the amount of CTGF mRNA and protein may change in cancerous tissue during tumor development, and it is not known whether this is a growth advantage for cancer cells or rather the reaction from stromal cells in order to inhibit the proliferation and metastasis (Kikuchi et al. 2007). Therefore, CTGF being expressed by cancer cells or surrounding stromal cells became an important player in the tumor microenvironment and in the bidirectional communication between cells (Capparelli et al. 2012). The dual nature of this growth factor is emphasized by the consequences of its deregulation in various cancers. An elevated expression of *CTGF* has been correlated with more advanced disease and worse survival outcomes in breast, gastric, esophageal and thyroid cancers as well as in gliomas, whereas significantly lowered level of CTGF protein has been detected in advanced, poorly differentiated colorectal tumors, in nasopharyngeal carcinoma and in NSCLCs and was associated with poor prognosis (Chang et al. 2004; Lin et al. 2005; Chen et al. 2007a; Liu et al. 2008; Zhou et al. 2009; Cui et al. 2011; Edwards et al. 2011; Zhen et al. 2013; Zhu et al. 2015).

In the present study, we demonstrated significantly lowered levels of CTGF transcript and protein in NSCLC cell lines (A549, Calu-1) and in lung cancerous tissues obtained from 98 patients with NSCLC. These data are consistent with the results of previous studies, showing that reduced expression of *CTGF* occurs in NSCLC (Chang et al. 2004; Chien et al. 2006; Chen et al. 2007a). We found lower levels of CTGF mRNA and protein in A549 cells than in Calu-1 cells. This in part reflects the findings of Chang et al. (2004) who reported that A549 cell line, as the most invasive among other investigated ADC cells, express a very low or even undetectable level of CTGF protein. The authors concluded that *CTGF* expression is inversely associated with an invasive phenotype of lung ADC cells. Nonetheless, both A549 and Calu-1 cell lines are characterized as highly invasive and Calu-1 was shown to possess greater invasive capability (Kumarswamy et al. 2012). Therefore, we cannot draw the same conclusion; however, it is important to notice that those cells belong to different histological subtypes of NSCLC.

In our work, we used both Western blot and immunohistochemistry techniques to establish the amount of CTGF protein in investigated patients’ specimens, while earlier studies performed immunohistochemical staining alone (Chang et al. 2004; Chien et al. 2006; Chen et al. 2007a). Our results are consistent with those previously described, as in analyzed specimens we did not detect CTGF protein in ADC cells and SCC cells presented no or only weak cytoplasmic staining pattern. However, the same as Chang et al. we found strong CTGF immunoreactivity in macrophages, stromal fibroblast and normal epithelial cells located within the tumor field. This may explain Western blot results, where in cancerous tissues we were able to detect CTGF protein. It is not know how the presence of this protein in tumor-infiltrating macrophages and stromal fibroblasts may influence the development of NSCLC. In high-grade serous ovarian tumors, where CTGF promotes migration and peritoneal adhesion of cancer cells, high amount of CTGF protein was detected in cancer-related stroma compared with matched cancer epithelial cells, whereas in breast cancer, high expression of *CTGF* in tumor cells but not in stromal cells had significant clinical relevance (Moran-Jones et al. 2015; Zhu et al. 2015).

Although Chang et al. (2004) found that lowered CTGF protein content was significantly associated with a higher grade of lymph node metastasis, larger tumor size and more advanced stage of cancer, we were not able to note this in our study. Perhaps that is because we did not divide patients into groups with lower or higher grade of *CTGF* expression in cancerous tissues. However, using RT-qPCR, Western blotting and immunohistochemistry we demonstrated that the great majority of NSCLC tumors express significantly lower levels of *CTGF* than paired normal lung tissues, and this phenomenon is correlated with various clinicopathological features. Even if the differences in *CTGF* mRNA and protein amounts between investigated tissues did not reach statistical significance among some groups, the decline trend in cancerous specimens was sustained. Moreover, we showed that the amount of CTGF protein in cancerous tissue is associated with histological type of LC, with the lowest content in ADC. Therefore, those results highlight the need to identify what role does CTGF play in NSCLC development.

The involvement of this growth factor in angiogenesis and in the metastatic phenotype of various malignancies is crucial and however still remains elusive. Yang and coworkers pinpointed CTGF as a major angiogenic inducer in prostate stromal cells. An elevated expression of *CTGF* in carcinoma-associated reactive stroma promoted the growth of LNCaP cells and increased microvessel density in nude mice (Yang et al. 2005). Furthermore, CTGF promoted the growth, motility and migration of glioblastoma multiforme cells, stimulated neovascularization and enhanced the ability of those cells to form tumors in mice (Yin et al. 2010). The amount of secreted and cell-associated CTGF protein was substantially increased in breast cancer cells exposed to hypoxic conditions, and CTGF was shown to be involved in the regulation of matrix metalloproteinases (MMPs) as well as their tissue inhibitors (TIMPs; Shimo et al. 2001; Kondo et al. 2002). The authors concluded that CTGF is the major factor promoting angiogenesis in vitro and in vivo and contributing to the invasion of breast cancer cells (Kondo et al. 2002). Accordingly, knockdown of *CTGF* expression significantly decreased the migration and an invasion rate of gastric cancer cell lines via reduction of *MMPs* expression level (Jiang et al. 2011). Further studies are needed to determine the impact of CTGF on *MMPs* and *TIMPs* expression and on the ECM rearrangement in NSCLC. On the contrary, CTGF was shown to bind the most abundant variant of vascular endothelial growth factor—VEGF 165, and thus inhibits its angiogenic properties (Inoki et al. 2002; Hashimoto et al. 2002). Forced expression of *CTGF* significantly lowered VEGF mRNA and protein levels in NSCLC cell lines and inhibited their invasion and metastasis in mouse xenograft tumor model (Chang et al. 2006). Further experiments indicated that CTGF exerts an anti-angiogenic effect in NSCLC by the reduction of hypoxia-inducible factor 1α (HIF-1α) protein stability (Chang et al. 2006). HIF-1α is recognized as a main transcription factor regulating *VEGF* expression (Ziello et al. 2007). During our work, we also observed the decreased HIF-1α protein amount in NSCLC cells, after an elevation of *CTGF* expression by TSA and 5-dAzaC treatment (data not shown). However, this was rather due to the destabilizing effect of those compounds on HIF-1α protein than the influence of CTGF itself (Liang et al. 2006). The development of solid tumors is often accompanied by hypoxic conditions that result in the accumulation of HIF-1α, which alters gene transcription, enhancing glucose uptake, glycolysis, oxygen transport and angiogenesis (Webb et al. 2009). Therefore, an elevation of the *CTGF* expression could be of benefit at least in NSCLC, as in other malignancies CTGF was shown to be a hypoxia-responsive gene leading to an enhancement of cancer cells invasion (Kondo et al. 2002, 2006; Braig et al. 2011; Eguchi et al. 2013).

The results of our work show that the expression of *CTGF* in NSCLC can be epigenetically regulated. Incubation of A549 and Calu-1 cells with 5-dAzaC or with TSA significantly increased CTGF mRNA and protein levels. The same effect was observed for Beas-2B cells. Previously, several studies have also established the role of epigenetic mechanisms in the regulation of *CTGF* expression in other cancers. Disturbances of the DNA methylation pattern within CpG islands of *CTGF* led to its aberrant expression in ovarian cancer and in precursor B-cell acute lymphoblastic leukemias (Kikuchi et al. 2007; Welch et al. 2013). Additionally, the transcription of *CTGF* was shown to be regulated by TSA in mouse renal cells and in skin fibroblasts obtained from patients with systemic sclerosis (Hemmatazad et al. 2009; Komorowsky et al. 2009). The epigenetic regulation of *CTGF* was also determined in hepatomas (Chiba et al. 2004). However, to the best of our knowledge, this is the first report indicating 5-dAzaC and TSA-induced over-expression of *CTGF* in NSCLC cells, although our findings suggest that 5-dAzaC induces the expression of *CTGF* in A549, Calu-1 and Beas-2B cells without altering the methylation status of its regulatory region. So, the mechanism of action is indirect and different than presented in ovarian cancer cell lines and tissues, where loss of *CTGF* expression was caused by hypermethylation of CpG islands and restored by 5-dAzaC (Kikuchi et al. 2007). Moreover, treatment of ovarian cancer cell lines with TSA alone had no effect on the expression level of *CTGF*, whereas in A549 and Calu-1 cells it significantly induced CTGF mRNA and protein content, suggesting that down-regulation of this gene in NSCLC cells and in ovarian cancer cells has distinct molecular basis. *CTGF* was identified as a functional target of several transcription factors and micro-RNAs which expression can be either epigenetically regulated (Chowdhury and Chaqour 2004; Chuang and Jones 2007; Komorowsky et al. 2009; Kubota and Takigawa 2015; Tian et al. 2015; Guo et al. 2015). Therefore, mechanisms other than direct DNA methylation in the regulatory region of *CTGF* may be responsible for its silencing in NSCLC cell lines.

Nonetheless, in our study we determined that the aberrant methylation of *CTGF* occurs in NSCLC tumors compared with histopathologically unchanged tissues. Although results were not statistically significant, we performed preliminary analysis for the presence of transcription factors binding sites in our *CTGF* regulatory sequence using JASPAR database and we found potential binding sites for several transcription factors (HES7, ZIC4; SP8, SP2) (Mathelier et al. 2015). However, their role in the regulation of *CTGF* expression in NSCLC needs to be investigated and the precise mechanisms by which 5-dAzaC and TSA induce the expression of *CTGF* in NSCLC cells need to be further established.

An application of tested compounds (5-dAzaC and TSA) could be of benefit in NSCLC, at least for two reasons: not only they increased the amount of CTGF transcripts and protein in NSCLC cell lines, but they also contributed to the reduction in cell proliferation. Cheng et al. (2004, 2006) reported that an elevated expression level of *CTGF* correlates with lower invasive and metastatic ability of lung ADC cells, in vitro and in vivo, in a mouse model. Nonetheless, it did not disturb the growth of those cells (Chang et al. 2004). However, another study by Chien et al. (2006) revealed that both an over-expression of *CTGF* or treatment with purified CTGF protein suppressed the growth of SCC and LCC cells. This raised the question whether CTGF actions are related to the specific histological subtype of NSCLC.

In the present study, we showed that both 5-dAzaC and TSA were able to reduce NSCLC cells proliferation in a p53-independent manner, as A549 cells possess a wild-type *TP53* and both alleles of *TP53* are deleted in Calu-1 cells. These results are consistent with previous findings. In melanoma cells, TSA was reported to induce growth arrest and apoptosis without the involvement of p53 protein (Peltonen et al. 2005) and several lines of evidence have indicated that both wild-type and *TP53*-deficient cells are very sensitive to 5-dAzaC (Nieto et al. 2004; Liu et al. 2012).

The potent antitumor activity of TSA against NSCLC cells was confirmed by Mukhopadhyay and coworkers. TSA evoked a tenfold greater growth inhibition of NSCLC cell lines in comparison with normal lung fibroblasts (Mukhopadhyay et al. 2006). Moreover, it is known that TSA presents a greater specificity for cancer cells (compared with normal counterparts) than other HDAC inhibitors (Chang et al. 2012). This feature allows it to be applied at low and apparently non-toxic doses. Indeed, we did not observe the cytotoxic effect of TSA on A549 and Calu-1 cells at an investigated range of concentrations (30–300 nM). Because the reactivation of silenced genes expression by DNMTs inhibitors may open the way for new treatment strategies, 5-dAzaC has been extensively studied in NSCLC with promising results (Momparler 2013). In our work, we showed that 5-dAzaC at concentrations of 10 and 15 µM was able to inhibit A549 and Calu-1 cells proliferation without the significant reduction in cell viability.

In conclusion, our study demonstrates that CTGF transcript and protein levels in lung cancerous tissues from NSCLC patients are significantly reduced compared with matched normal control specimens and that the expression of *CTGF* in NSCLC can be epigenetically regulated and restored. Further studies are needed to determine whether *CTGF* targeting would be of benefit during NSCLC therapy.

## Electronic supplementary material

Below is the link to the electronic supplementary material.

Supplementary material 1 (DOCX 3656 kb)

Supplementary material 2 (DOCX 472 kb)

Supplementary material 3 (DOCX 98 kb)

Supplementary material 4 (DOCX 663 kb)

Supplementary material 5 (DOCX 16 kb)

Supplementary material 6 (DOCX 15 kb)

Supplementary material 7 (DOCX 15 kb)
