# Supplementary material for: Decreased expression of connective tissue growth factor in non-small cell lung cancer is associated with clinicopathological variables and can be restored by epigenetic modifiers
Source: J Cancer Res Clin Oncol. 2016 Jul 8;142(9):1927–46. doi: 10.1007/s00432-016-2195-3 (PMC4978771; doi:10.1007/s00432-016-2195-3)
Supplement: Supplementary file 1 — Supplementary material 1 (DOCX 3656 kb) [file 432_2016_2195_MOESM1_ESM.docx]

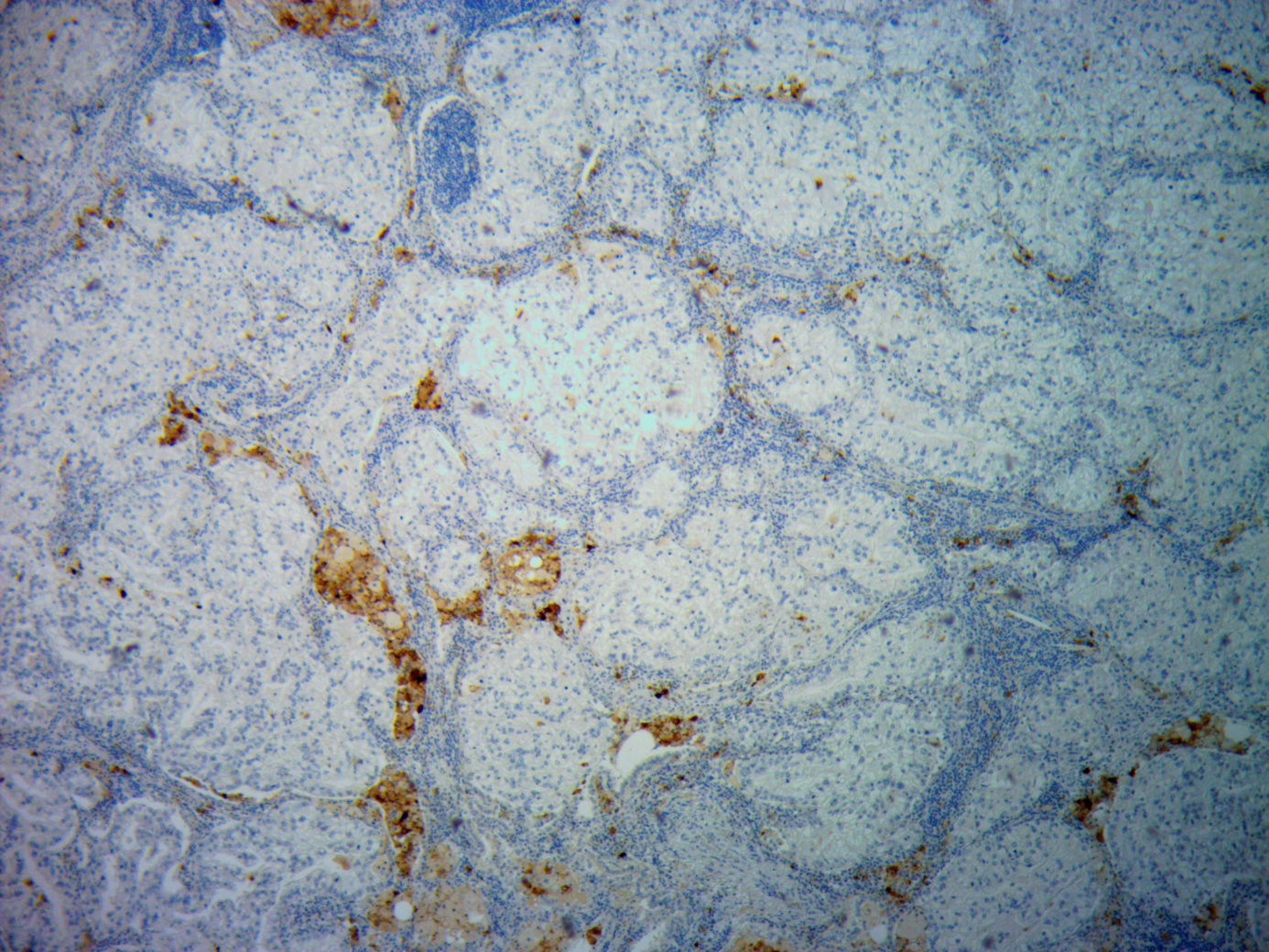


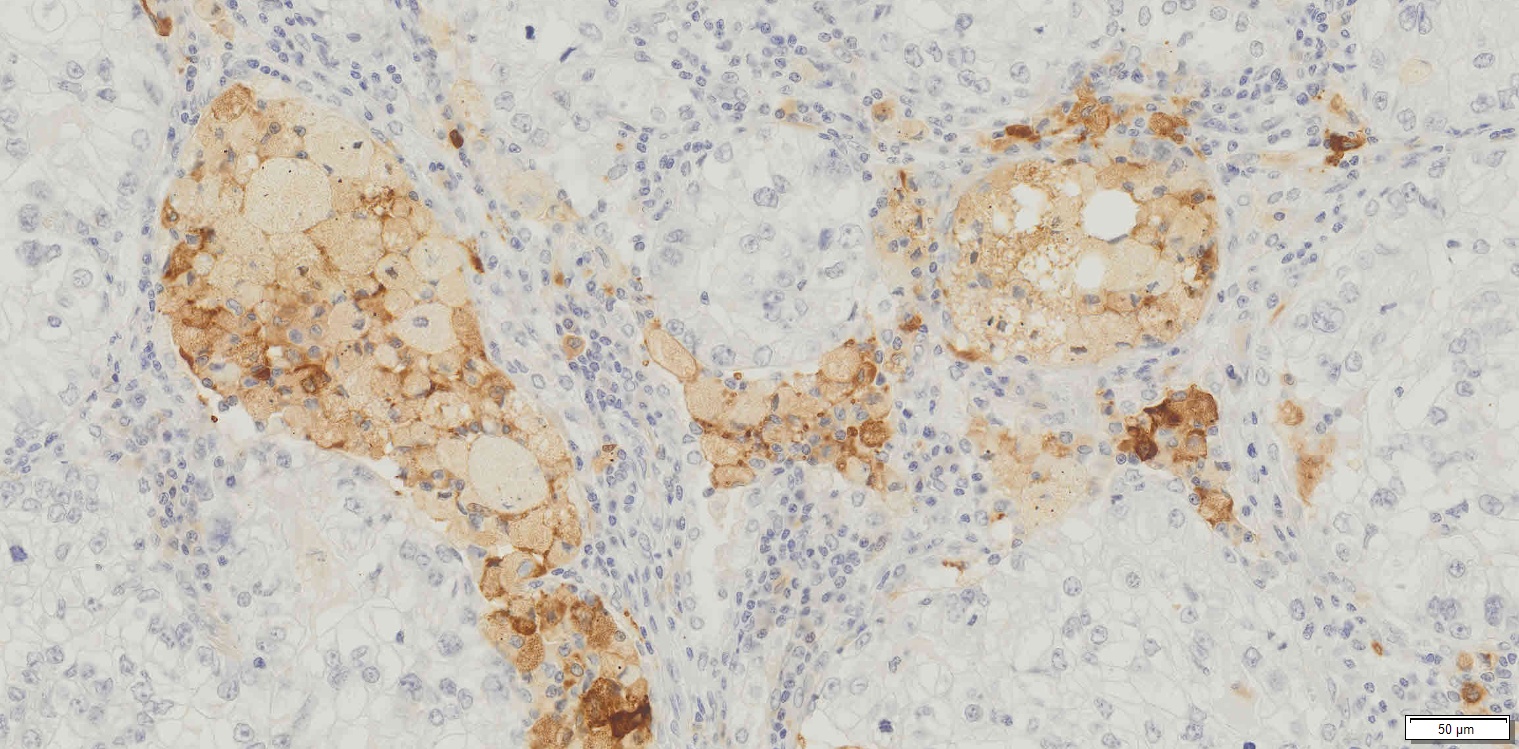


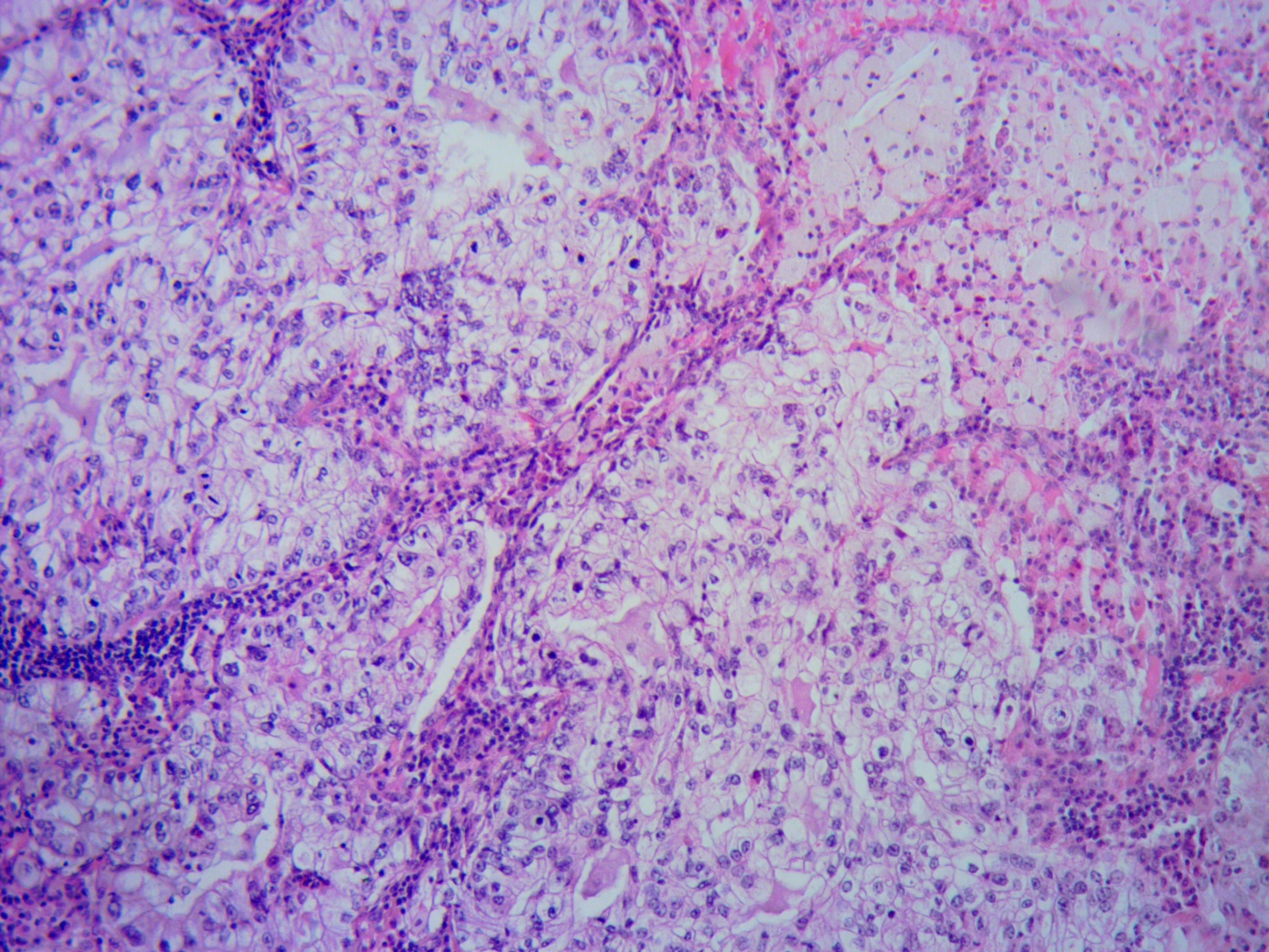


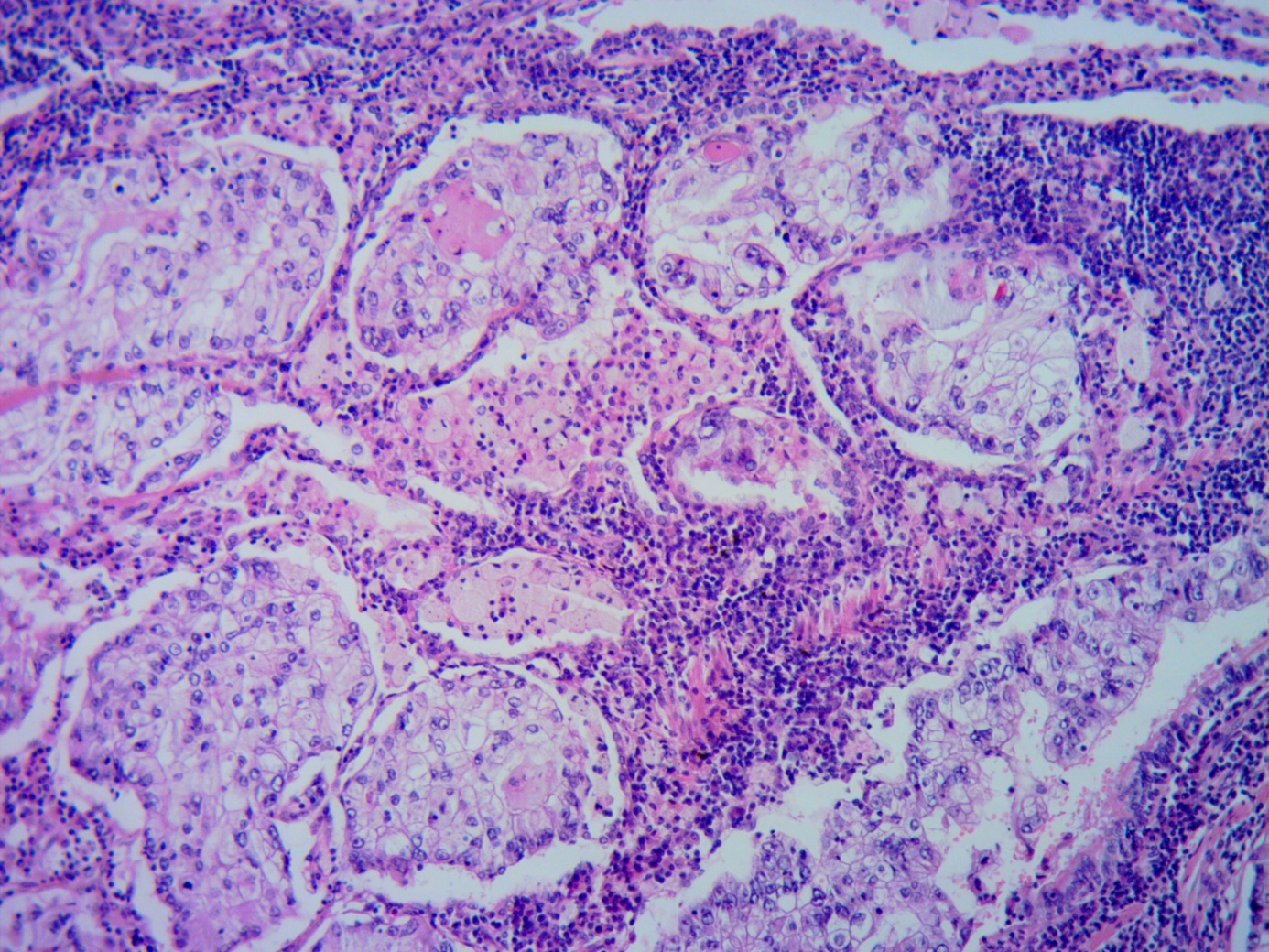


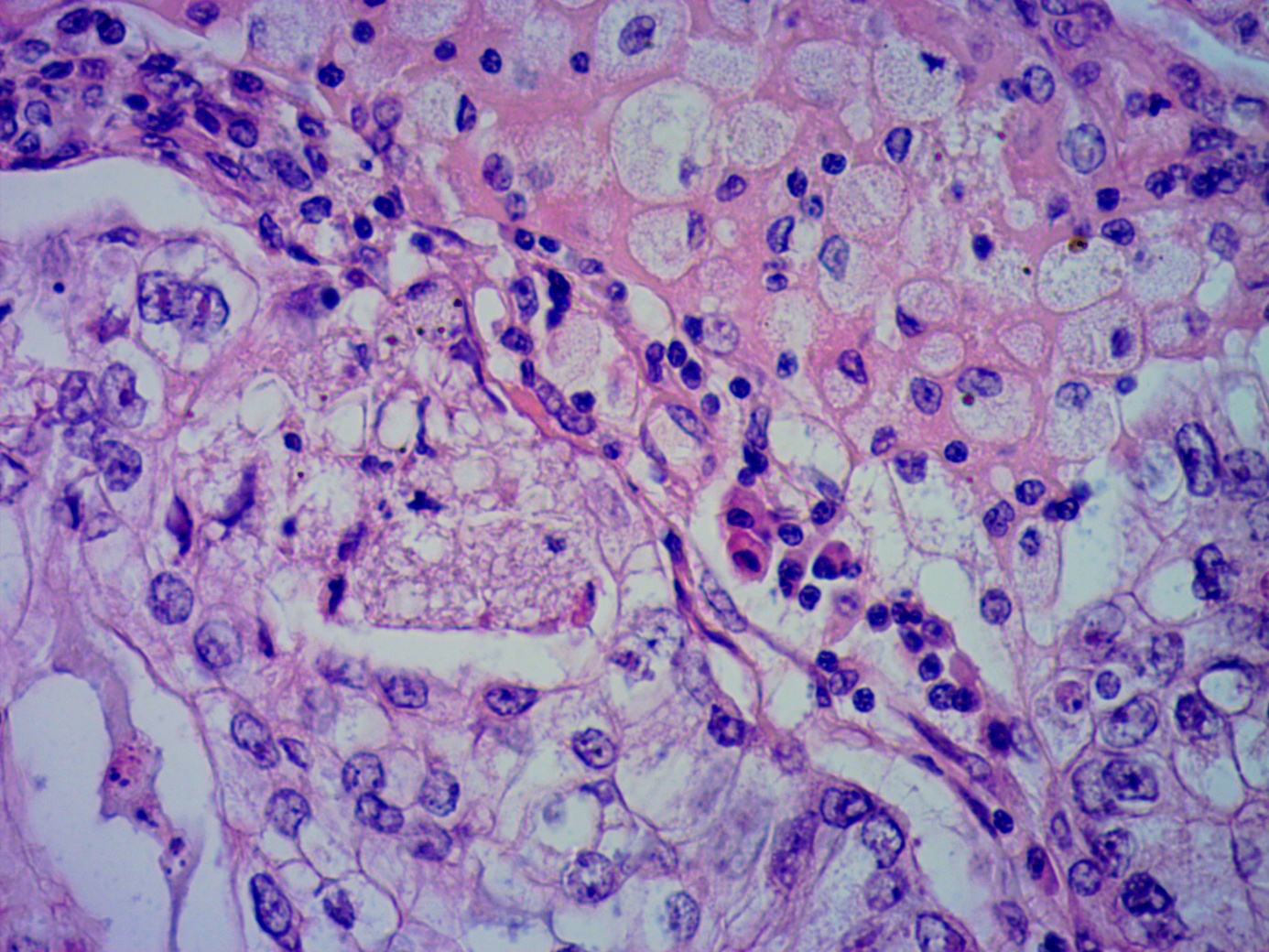


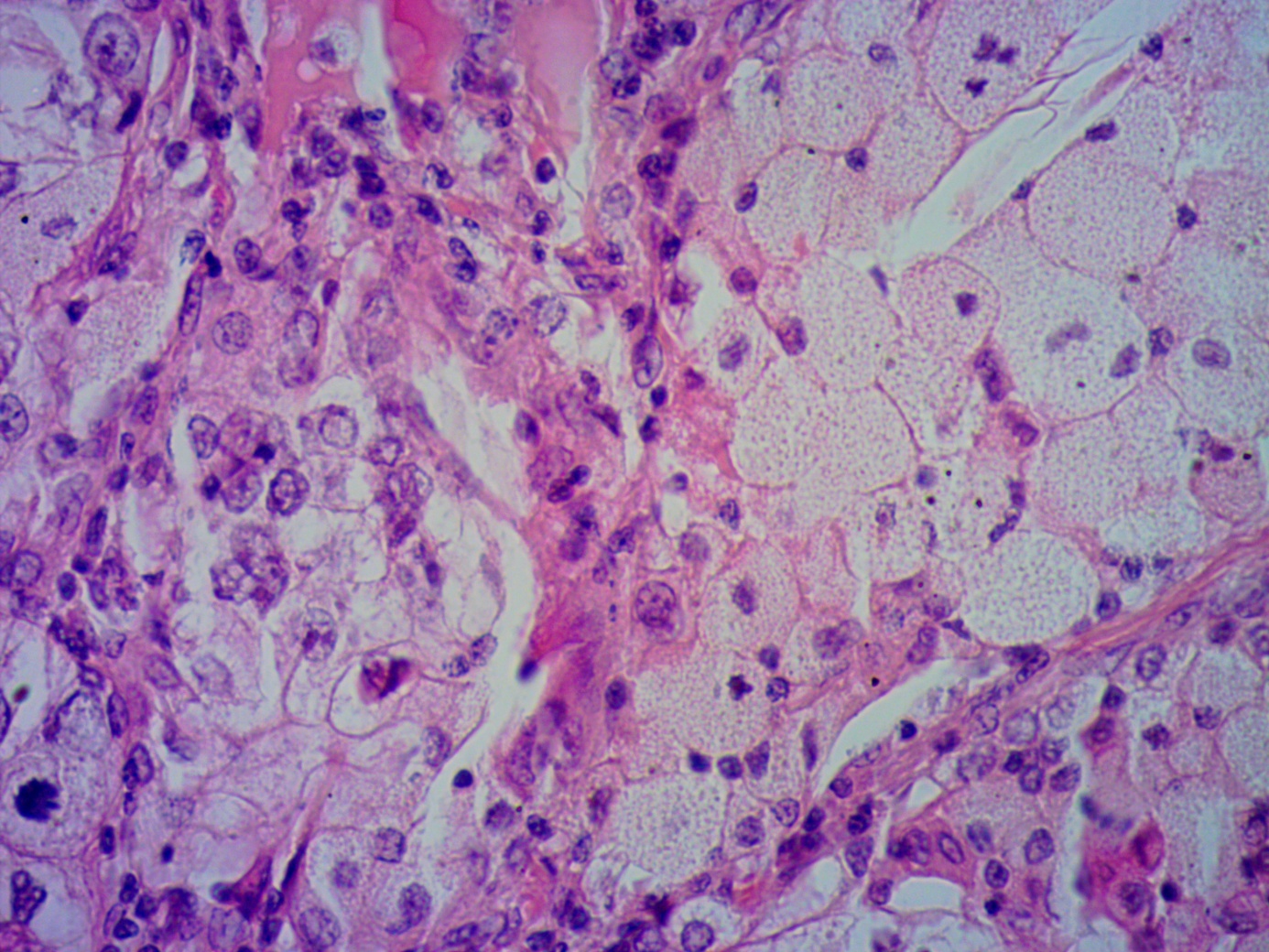


**Supplementary Figure 1** **Representative CTGF immunohistochemistry results and hematoxylin and eosin staining of formalin-fixed, paraffin-embedded clinical tissue specimen**

**a**, **b** Immunohistochemistry images of selected ADC specimen with ADC cells showing no staining for CTGF. Positive immunoreactivity is indicated by the brown staining and restricted to macrophages. **c**, **d**, **e**, **f** Representative hematoxylin and eosin staining of different areas of selected ADC specimen showing tumour-associated macrophages. Original magnifications x40 (**a)**,
x100 (**c,** **d**), x200 (**b**), x400 (**e, f**)
