# Supplementary material for: Decreased expression of connective tissue growth factor in non-small cell lung cancer is associated with clinicopathological variables and can be restored by epigenetic modifiers
Source: J Cancer Res Clin Oncol. 2016 Jul 8;142(9):1927–46. doi: 10.1007/s00432-016-2195-3 (PMC4978771; doi:10.1007/s00432-016-2195-3)
Supplement: Supplementary file 2 — Supplementary material 2 (DOCX 472 kb) [file 432_2016_2195_MOESM2_ESM.docx]

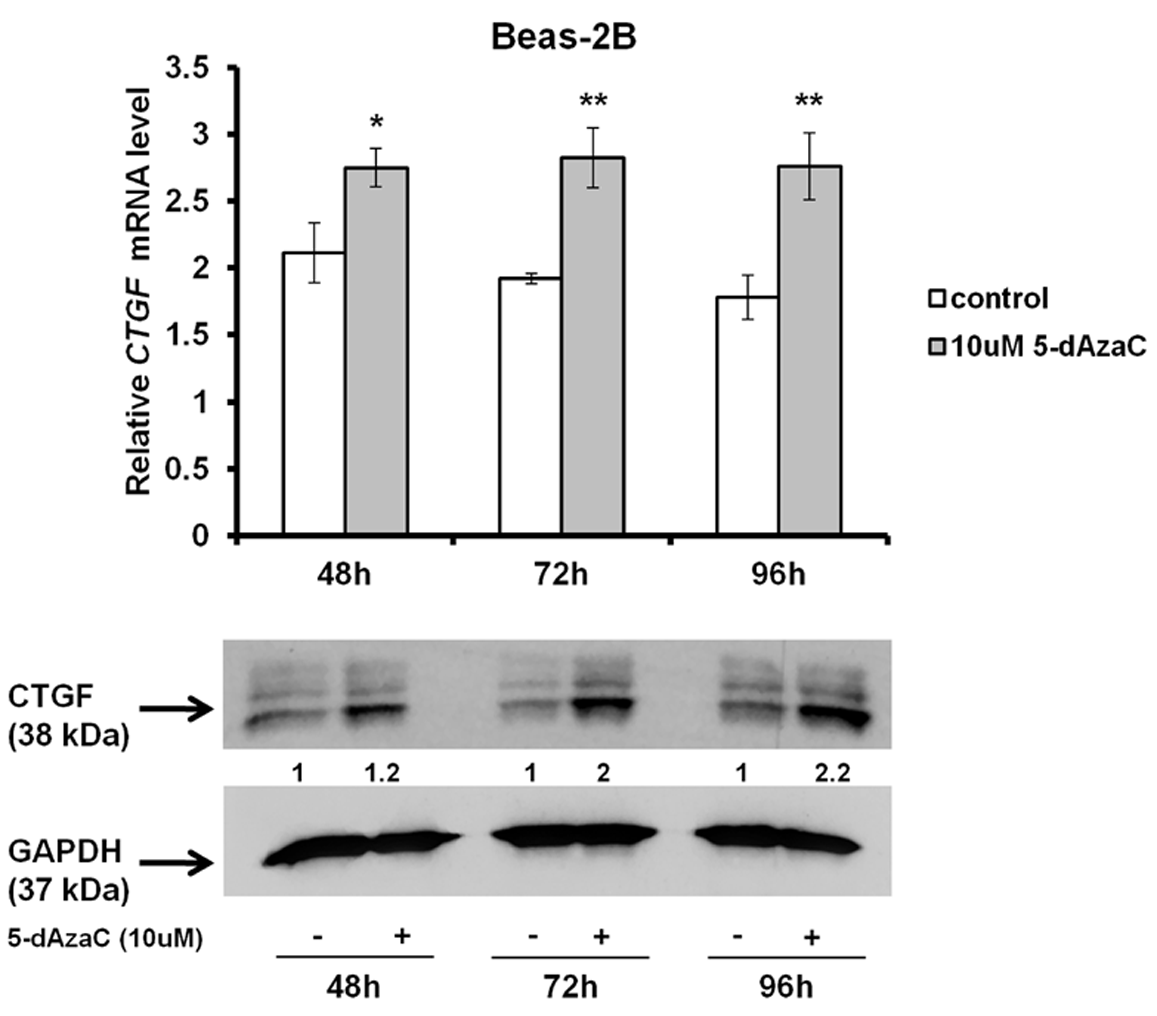


**Supplementary Figure 2 The effect of 5-dAzaC on CTGF transcript and protein levels in
Beas-2B cell line**

Cells were cultured for 48, 72 and 96 h in the absence or in the presence of 5-dAzaC at a concentration of 10 μM. Next, cells were used for total RNA and protein isolation. RT-qPCR results were standardized by the geometric mean of *PBGD* and *hMRPL19* cDNA levels. *CTGF* transcript levels are presented as multiplicity of the respective controls. Each sample was determined in triplicate and results represent means ± SD from three experiments (* p < 0.05; ** p < 0.001). 30 μg of protein isolated from each sample was separated by 12% SDS-PAGE. After transfer, a nitrocellulose membrane was immunoblotted with goat polyclonal anti-CTGF Ab, followed by rabbit anti-goat HRP-conjugated Ab. Membranes were stripped and reblotted with rabbit polyclonal anti-GAPDH Ab and goat anti-rabbit HRP-conjugated Ab. The band densitometry readings were normalized to GAPDH loading control. The ratio of CTGF to GAPDH protein for cells incubated in the absence of 5-dAzaC for each period of time was assumed to be 1
