# Supplementary material for: Decreased expression of connective tissue growth factor in non-small cell lung cancer is associated with clinicopathological variables and can be restored by epigenetic modifiers
Source: J Cancer Res Clin Oncol. 2016 Jul 8;142(9):1927–46. doi: 10.1007/s00432-016-2195-3 (PMC4978771; doi:10.1007/s00432-016-2195-3)
Supplement: Supplementary file 3 — Supplementary material 3 (DOCX 98 kb) [file 432_2016_2195_MOESM3_ESM.docx]

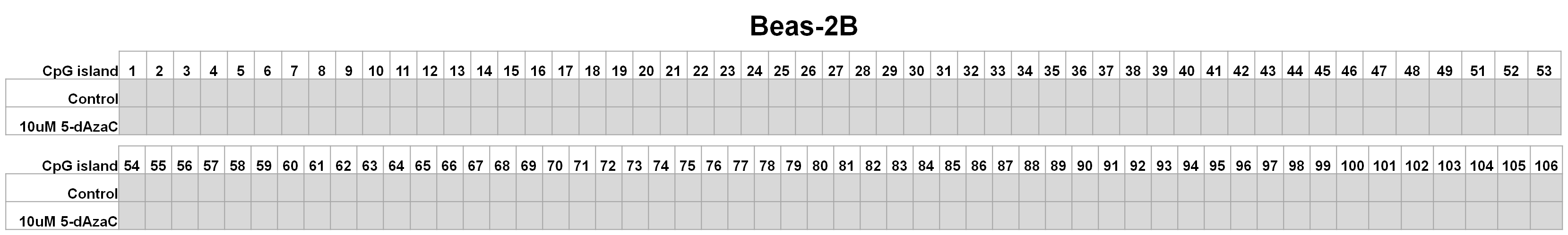


**Supplementary Figure 3 DNA methylation assessment of *CTGF* regulatory region by bisulfite sequencing in Beas-2B cell line**

Beas-2B cells were cultured for 96 h either in the absence or in the presence of 5-dAzaC at a concentration of 10 μM. Then, cells were used for genomic DNA isolation, followed by bisulfite conversion of cytosine to uracil. The CpG rich region containing 106 CpG dinucleotides was then amplified by a pair of primers complementary to the bisulfite-DNA modified sequence (Supplementary table 3). The PCR products were purified with subsequent cloning into a plasmid vector. Plasmid DNA isolated from five bacterial clones was used for commercial sequencing. The results of bisulfite sequencing were assessed and presented using BiQ analyzer software and BDPC web server. Grey boxes represent unmethylated CpG dinucleotides
