# Supplementary material for: Decreased expression of connective tissue growth factor in non-small cell lung cancer is associated with clinicopathological variables and can be restored by epigenetic modifiers
Source: J Cancer Res Clin Oncol. 2016 Jul 8;142(9):1927–46. doi: 10.1007/s00432-016-2195-3 (PMC4978771; doi:10.1007/s00432-016-2195-3)
Supplement: Supplementary file 4 — Supplementary material 4 (DOCX 663 kb) [file 432_2016_2195_MOESM4_ESM.docx]

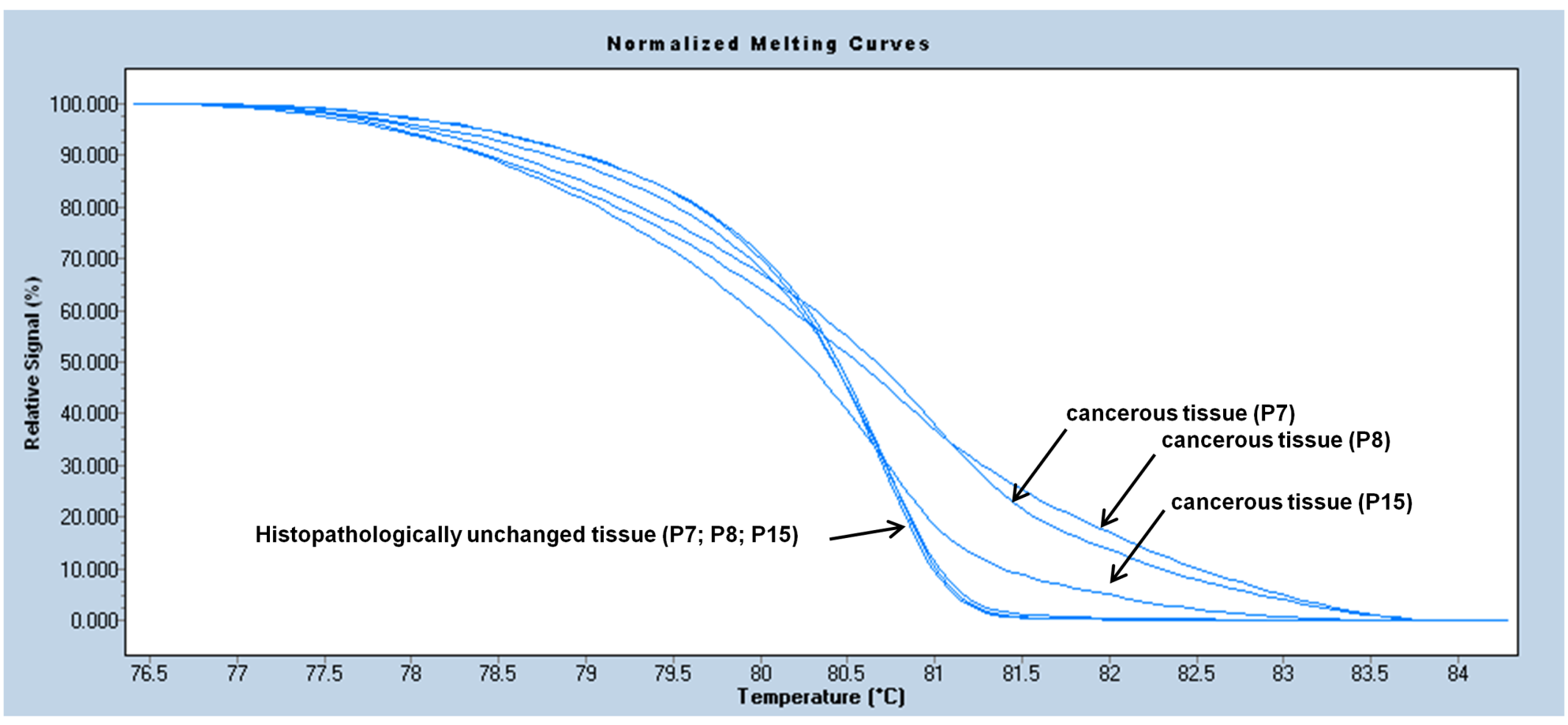


**Supplementary Figure 4 Representative profiles of high resolution melting curves used for the assessment of different DNA methylation patterns of *CTGF* regulatory region by MS-HRM analysis in primary tissue samples from NSCLC patients**

The CTGF region containing 15 CpG dinucleotides located at Chr6: 132271312 – 132271581 (according to UCSC GRCh37/hg19) was amplified by a pair of primers complementary to the bisulfite DNA-modified sequence (Supplementary table 3). HRM methylation analysis was performed using Light Cycler®480 Gene Scanning software, Roche Diagnostics GmbH (Mannheim, Germany). Different HRM profiles for histopathologically unchanged tissues and lung cancerous tissues for three patients are presented
