# Supplementary material for: Decreased expression of connective tissue growth factor in non-small cell lung cancer is associated with clinicopathological variables and can be restored by epigenetic modifiers
Source: J Cancer Res Clin Oncol. 2016 Jul 8;142(9):1927–46. doi: 10.1007/s00432-016-2195-3 (PMC4978771; doi:10.1007/s00432-016-2195-3)
Supplement: Supplementary file 5 — Supplementary material 5 (DOCX 16 kb) [file 432_2016_2195_MOESM5_ESM.docx]

**Supplementary table 1 Differences in *CTGF* transcript levels in lung cancer and corresponding histopathologically unchanged tissues from NSCLC patients including TNM classification**

| **Variables** | **Number of cases** | **cancerous tissues** | **histopathologically unchanged tissues** | **p value**  ***CTGF* mRNA** |
| --- | --- | --- | --- | --- |
|  |  | Mean±SD | Mean±SD |  |
| **Total no. of patients** | 98 | 2.58 ± 0.41 | 3.12±0.4 | < 0.0000001 |
| **Tumor size** |  |  |  |  |
| Tis | 5 | 2.45 ± 0.55 | 3.13 ± 0.50 | 0.08 |
| T1a | 7 | 2.71 ± 0.29 | 3.14 ± 0.39 | 0.04 |
| T1b | 9 | 2.65 ± 0.56 | 3.14 ± 0.26 | 0.03 |
| T2a | 46 | 2.53 ± 0.36 | 3.08 ± 0.39 | 0.0000001 |
| T2b | 11 | 2.62 ± 0.19 | 3.22 ± 0.38 | 0.0001 |
| T3 | 15 | 2.49 ± 0.50 | 3.09 ± 0.52 | 0.003 |
| T4 | 5 | 2.94 ± 0.49 | 3.23 ± 0.34 | 0.3 |
| **Lymph node metastasis** |  |  |  |  |
| N0 | 54 | 2.56 ± 0.40 | 3.04 ± 0.40 | 0.0000001 |
| N1 | 34 | 2.67 ± 0.40 | 3.21 ± 0.40 | 0.0000001 |
| N2 | 10 | 2.35 ± 0.42 | 3.19 ± 0.28 | 0.00006 |
| N3 | _ | _ | _ | _ |
| **Distant metastasis** |  |  |  |  |
| M0 | 95 | 2.57 ± 0.41 | 3.12 ± 0.40 | 0.0000001 |
| M1a | 3 | 2.86 ± 0.43 | 2.99 ± 0.22 | _ |
| M1b | _ | _ | _ | _ |

*CTGF* transcript levels were standardized by the geometric mean of *PBGD* and *hMRPL19* cDNA levels. Results were expressed as decimal logarithm of multiples of cDNA copies in the calibrator. The normality of observed patient data distribution was assessed by Shapiro-Wilk test and parametric unpaired, two-tailed *t*-test was used to compare the mean values. p < 0.05 was considered as statistically significant
