# Supplementary material for: Decreased expression of connective tissue growth factor in non-small cell lung cancer is associated with clinicopathological variables and can be restored by epigenetic modifiers
Source: J Cancer Res Clin Oncol. 2016 Jul 8;142(9):1927–46. doi: 10.1007/s00432-016-2195-3 (PMC4978771; doi:10.1007/s00432-016-2195-3)
Supplement: Supplementary file 6 — Supplementary material 6 (DOCX 15 kb) [file 432_2016_2195_MOESM6_ESM.docx]

**Supplementary table 2 Differences in CTGF protein levels in lung cancerous and corresponding histopathologically unchanged tissues from NSCLC patients including TNM classification**

| **Variables** | **Number of cases** | **cancerous tissues** | **histopathologically unchanged tissues** | **p value**  **CTGF protein** |
| --- | --- | --- | --- | --- |
|  |  | Mean±SD | Mean±SD |  |
| **Total no. of patients** | 98 | 2.79 ± 0.32 | 3.06 ± 0.34 | < 0.0000001 |
| **Tumor size** |  |  |  |  |
| Tis | 5 | 2.98 ± 0.36 | 3.18 ± 0.54 | 0.6 |
| T1a | 7 | 2.75 ± 0.38 | 3.11 ± 0.44 | 0.1 |
| T1b | 9 | 2.94 ± 0.27 | 3.26 ± 0.23 | 0.02 |
| T2a | 46 | 2.70 ± 0.29 | 3.06 ± 0.33 | 0.000002 |
| T2b | 11 | 2.80 ± 0.46 | 2.79 ± 0.44 | 0.9 |
| T3 | 15 | 2.83 ± 0.23 | 3.18 ± 0.25 | 0.0007 |
| T4 | 5 | 2.97 ± 0.36 | 3.06 ± 0.27 | 0.7 |
| **Lymph node metastasis** |  |  |  |  |
| N0 | 54 | 2.78 ± 0.33 | 3.09 ± 0.31 | 0.000004 |
| N1 | 34 | 2.80 ± 0.33 | 3.03 ± 0.39 | 0.01 |
| N2 | 10 | 2.79 ± 0.26 | 3.16 ± 0.41 | 0.03 |
| N3 | _ | _ | _ | _ |
| **Distant metastasis** |  |  |  |  |
| M0 | 95 | 2.78 ± 0.32 | 3.09 ± 0.35 | < 0.0000001 |
| M1a | 3 | 2.80 ± 0.36 | 2.85 ± 0.21 | _ |
| M1b | _ | _ | _ | _ |

The amount of proteins detected by Western blotting was presented as the decimal logarithm of CTGF to GAPDH band optical density ratio. The normality of observed patient data distribution was assessed by Shapiro-Wilk test and parametric unpaired, two-tailed *t*-test was used to compare the mean values. p < 0.05 was considered as statistically significant
