# Supplementary material for: Decreased expression of connective tissue growth factor in non-small cell lung cancer is associated with clinicopathological variables and can be restored by epigenetic modifiers
Source: J Cancer Res Clin Oncol. 2016 Jul 8;142(9):1927–46. doi: 10.1007/s00432-016-2195-3 (PMC4978771; doi:10.1007/s00432-016-2195-3)
Supplement: Supplementary file 7 — Supplementary material 7 (DOCX 15 kb) [file 432_2016_2195_MOESM7_ESM.docx]

**Supplementary table 3 Primer sequences used for RT-qPCR analysis and bisulfite sequencing**

| **Gene** | **Sequence (5’-3’)** | **ENST/ENSG number/**  ***www.ensembl.org*/** | **Product size (bp)** |
| --- | --- | --- | --- |
| **Primers for RT-qPCR** | | | |
| *CTGF* | F: TTACCAATGACAACGCCTCCT  R: CTTTTTGCCCTTCTTAATGTTCT | ENST00000367976 | 104 |
| *PBGD* | F: GCCAAGGACCAGGACATC  R: TCAGGTACAGTTGCCCATC | ENST00000278715 | 160 |
| *hMRPL19* | F: ACTTTATAATCCTCGGGTC  R: ACTTTCAGCTCATTAACAG | ENST00000393909 | 171 |
| **Primers for bisulfite sequencing of CpG island** | | | |
| *CTGF* | F: TGTAGGATTTTATTTAGTTTATTGG  R: ATCAAACATTAAAACACTCTCACA | ENSG00000118523 | 867 |
| **Primers for MS-HRM** | | | |
| *CTGF* | F: TGGGTTGTATGTTTTTGTGTAG  R: AAACACACAAACACCATATAAAAC | ENSG00000118523 | 270 |

F- forward

R- reverse
